# Gamma irradiation effects on optical, luminescent, and electrical properties of CeO_2_, Ho_2_O_3_, and Sm_2_O_3_ doped borophosphate glasses

**DOI:** 10.1038/s41598-026-63236-1

**Published:** 2026-09-05

**Authors:** I. S. Ali, Kh. Roumaih, M. A. Marzouk

**Affiliations:** 1 Higher Institute of Optics Technology, Heliopolis, Cairo, Egypt; 2 Reactor Physics Department, Nuclear Research Center, Egyptian Atomic Energy Authority, Cairo, 13759 Egypt; 3 Glass Research Department, National Research Centre, 33 EL Bohouth St. (Former EL Tahrir St.), Dokki, P.O.12622, Giza, Egypt

**Keywords:** Glass, Rare-earth, Oxides, Photoluminescence, Electrical conductivity, Gamma – irradiation, Materials science, Nanoscience and technology, Optics and photonics, Physics

## Abstract

A series of undoped and rare-earth oxide-doped borophosphate glasses (CeO_2_, Ho_2_O_3_, Sm_2_O_3_) was successfully synthesized via melt-quenching and annealing techniques, with x-ray diffraction confirming their amorphous nature through characteristic broad halos devoid of sharp crystalline peaks. Ultraviolet–visible (UV–Vis) absorption spectra of the undoped base glass revealed prominent UV bands at 265 nm and 330 nm before gamma irradiation, which persisted post-75 kGy exposure with only modest intensity reductions, indicating subtle radiation-induced modifications in defect states without profile disruption. CeO_2_—doped glasses exhibited three strong UV peaks at 220, 260, and 327 nm. At the same time, Ho^3+^-doped variants displayed a UV band at 295 nm alongside visible-NIR transitions at 418, 451, 482, 539, 644, 894, 1146, and 1944 nm from ^5^I_8_ ground state excitations; Sm_2_O_3_-doped glasses showed distinct f-f transitions at 354, 403, 470, 920, 1064, 1221, 1365, 1474, and 1520 nm. Photoluminescence analysis highlighted intrinsic features such as a 330 nm excitation and 408 nm emission in CeO_2_—doped glasses, three excitation peaks (350, 364, 387 nm) and emissions at 487 and 573 nm in Ho_2_O_3_-doped glasses, and seven excitation peaks (345–475 nm at λ_ex_ = 598 nm) in Sm_2_O_3_-doped glasses, all exhibiting characteristic intensity decreases post-irradiation. CIE 1931 chromaticity coordinates positioned CeO_2_—doped emissions in bluish-purple, Ho_2_O_3_-doped in green (0.275, 0.405 pre-irradiation), and Sm_2_O_3_ -doped in reddish-orange regions. Electrically, AC conductivity showed a slight decrease followed by an increase with rising temperature across compositions, DC conductivity displayed non-monotonic behavior with a minimum at 384 K, and real dielectric permittivity (ε′) was elevated at low frequencies due to space charge polarization. Overall, gamma irradiation induced only minor effects on optical and electrical properties, attributable to dopant-driven structural modifications like non-bridging oxygen formation and enhanced charge carrier pathways via modifier ions, underscoring the radiation resilience and potential of these glasses for luminescent, photonic, and radiation-resistant applications.

## Introduction 

Among the various types of glass host matrices, those that are classified as multi-former glasses stand out due to their composition, which typically includes more than one network-forming oxide. This unique characteristic enables these glasses to exhibit a broad spectrum of exceptional properties, making them highly suitable for a diverse array of innovative technological applications. By strategically selecting and tailoring the chemical composition of these multi-former glasses, researchers and material scientists can optimize their performance to meet specific needs. This adaptability allows for the enhancement of various attributes such as mechanical strength, thermal stability, and optical clarity, thereby paving the way for advancements in fields like telecommunications, electronics, and energy storage solutions.

Boro-phosphate glasses have been widely studied and recognized as one of the most prevalent types of glasses, primarily composed of two key glass-forming oxides: (B_2_O_3_) and phosphorus pento-oxide (P_2_O_5_). These innovative glasses offer numerous advantages, including a cost-effective synthesis process and the flexibility to be formed into various shapes. Additionally, they have the capacity to incorporate a diverse range of functional ions, atoms, or molecules, which enables them to fulfill multiple technological applications. Their versatility makes them suitable for use in multicolor lighting, scintillation detectors, infrared lasers, display devices, optical sensors, fiber amplifiers, bio probes, and various biomedical applications^1–4^.

Further, rare-earth-doped borophosphate glasses have garnered considerable attention in recent years, primarily due to their unique structural characteristics and their exceptional optical and electrical properties. The incorporation of rare earth ions, particularly those in the trivalent state, significantly contributes to the improvement of these properties^3,4^. These rare earth ions contribute to the glasses’ ability to manipulate light and electricity in ways that are not achievable with traditional materials. The distinctiveness of these glasses lies not only in their composition but also in the way the rare earth ions interact with the borophosphate matrix, leading to improved performance in various applications^3–5^.

The characteristics of borophosphate glasses are defined by a complex and adaptable structural framework that combines boron oxide (B_2_O_3_) and phosphorus oxide (P_2_O_5_) within a non-crystalline matrix. The essential structural components of these glasses are formed by tetrahedral phosphate (PO_4_) groups, which are linked together by bridging oxygens (B-O-P linkages) to borate units, including both borate (BO_3_ and BO_4_) structures. This intricate connectivity contributes to the unique properties and functionality of borophosphate glasses^4–6^.

This connectivity plays a vital role in shaping the overall network topology of borophosphate glasses, which in turn affects their physical and chemical properties. The addition of different modifiers, including alkali metal oxides and alkaline earth metal oxides, can substantially modify the glass structure. These modifiers introduce non-bridging oxygens (NBOs) into the network, which interrupt the polymerized chains of the borophosphate framework^4–8^. This disruption leads to significant changes in the glass’s characteristics, highlighting the importance of these modifiers in tailoring the material’s performance for specific applications.

The electrical properties of rare earth ion (RE^3+^)-doped glasses exhibit notable alterations in both conductivity and dielectric behavior, which can be attributed to the distinctive interactions that occur between the dopant ions and the glass matrix^9,10^. These interactions are essential in determining how the glass responds to electric fields, leading to variations in its overall electrical performance. As the rare earth ions integrate into the glass structure, they influence the movement of charge carriers and the material’s ability to store and dissipate electrical energy^9–13^. This phenomenon is particularly emphasized in studies that show how direct current (DC) conductivity exhibits Arrhenius-type behavior, indicating that the mechanisms of conduction are thermally activated^14,15^. These investigations demonstrate that as temperature increases, conductivity rises in a manner consistent with Arrhenius’ equation, suggesting that charge carriers acquire sufficient thermal energy to surmount potential barriers with rising temperatures^14,15^. With an increase in the concentration of rare earth ions (RE^3+^), there is generally an observed rise in the direct current conductivity (σ_dc_), accompanied by a decrease in activation energy. This trend indicates that there is enhanced ionic mobility within the glass network, allowing for more efficient charge transport^14,15^. Conversely, the incorporation of rare earth ions also affects the dielectric properties of the material, influencing both the real part (ε′) and the imaginary part (ε′′) of the dielectric constant^9–15^. These changes suggest a complex interplay between ionic mobility and dielectric behavior, highlighting the multifaceted impact that rare earth doping has on the overall electrical characteristics of the glass.

Moreover, alternating current (AC) conductivity (σ_ac_) demonstrates a power law behavior, suggesting that hopping conduction occurs among localized states within the glass matrix. From an optical perspective, glasses doped with rare earth ions (RE^3+^) display unique absorption and emission properties resulting from electronic transitions of these ions. The luminescence spectra typically show multiple emission lines that correspond to specific transitions, making them potentially valuable for laser applications^10–12^.

The intensity and placement of the emission bands in rare-earth-doped glasses can vary considerably depending on the concentration of the dopants and the specific composition of the glass. This variability underscores the critical need to optimize these parameters to achieve the desired optical performance. By carefully adjusting the doping concentration and tailoring the glass matrix, researchers can influence how effectively these materials absorb and emit light. Such optimization is essential for enhancing the functionality of the glasses in various applications, particularly in fields like photonics and laser technology, where precise control over optical properties is paramount. The interplay between doping levels and glass composition thus plays a vital role in determining the overall performance of these advanced materials.

Based on the previous introduction, the primary aim of the current study is to comprehensively evaluate the optical performance and electrical conductivity of rare-earth-doped borophosphate glasses, both in their pristine state and following exposure to a gamma irradiation dose of 75 kGy, thereby elucidating the radiation stability of glass and potential enhancements induced by doping and irradiation. This involves a detailed investigation of the UV–visible-near infrared (UV–Vis-NIR) absorption spectra to assess changes in electronic transitions, band gap characteristics, and defect-related absorption bands, alongside an examination of the photoluminescence (PL) behavior to probe emission properties, quenching effects, and radiative recombination mechanisms under irradiation-induced modifications. Complementing these optical analyses, the electrical properties are rigorously characterized through measurements of alternating current (AC) conductivity, direct current (DC) conductivity, and dielectric constant as a function of frequency and temperature, providing insights into charge carrier dynamics, ionic mobility, and polarization responses in the RE-doped glass matrix. Such multifaceted characterization not only reveals the interplay between rare -earth doping and gamma irradiation on the glass structure potentially leading to identify the promising applications of these glasses in radiation-resistant optical components, such as dosimeters, luminescent coatings for harsh environments, high-performance transparent electrodes in optoelectronic devices, and protective shields for space or nuclear applications where both optical clarity and electrical functionality must withstand ionizing radiation.

The current work demonstrates the successful preparations of Ce^3+^-, Ho^3+^-, and Sm^3+^-doped borophosphate glasses and establishes a comprehensive correlation between rare-earth doping, gamma irradiation, and their effects on the structural, optical, photoluminescence, and electrical properties. The results provide new insights into defect formation and energy transfer mechanisms, highlighting the potential of these glasses for radiation dosimetry, optical sensing, and radiation-resistant photonic devices. Future studies will focus on optimizing multi type dopants and evaluating long-term radiation stability to further enhance their multifunctional performance.

## Materials and methods

### Method of preparation

**Fig. 1:**
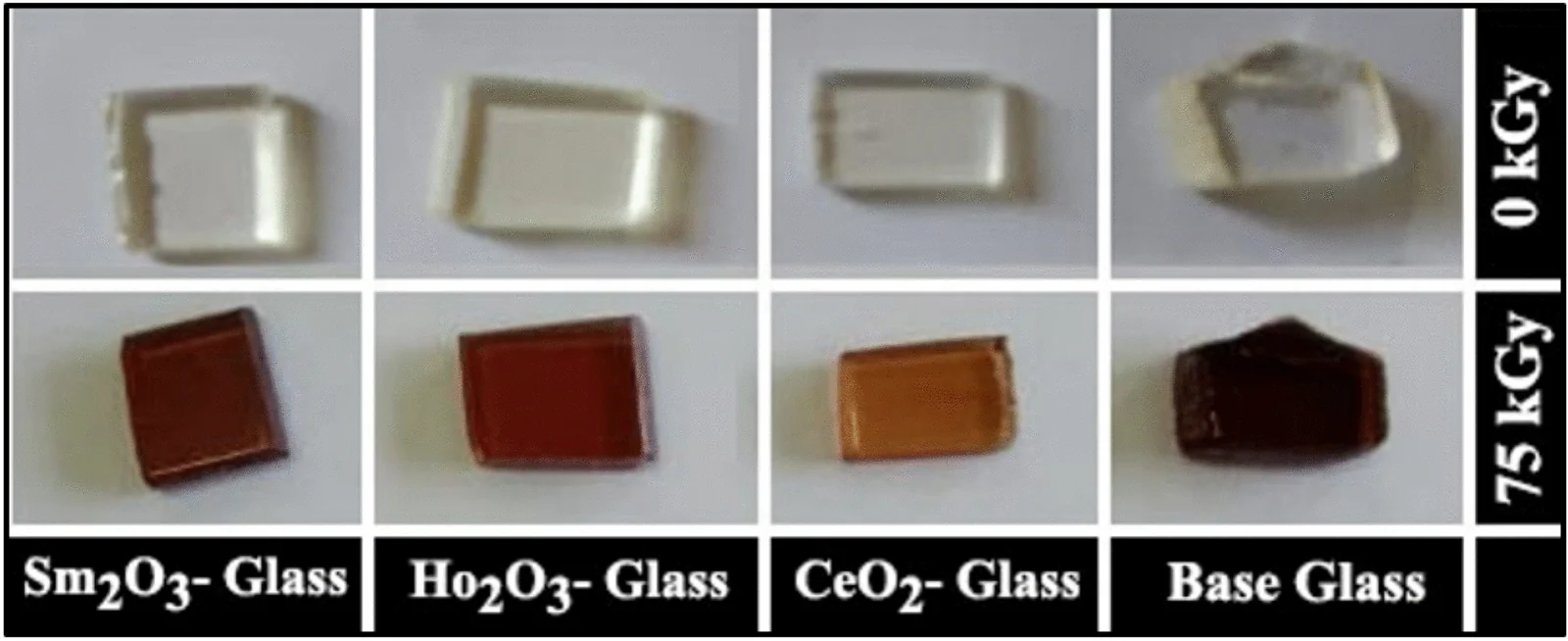
Photographic view of the prepared glass samples before and after gamma irradiation.

The undoped base alkali borophosphate glasses in addition incorporating rare earth oxide dopants from rare-earth oxides, specifically including Ho_2_O_3_, Sm_2_O_3_, and CeO_2_ as depicted in Table 1 Chemically pure (99.99%) NH_4_H_2_PO_4_, H_3_BO_3,_ Na_2_CO_3_, CaF_2_, Al_2_O_3_, Ho_2_O_3_, Sm_2_O_3_, and CeO_2_ from Sigma Aldrich were applied in glass preparations. The glasses were prepared by melting the precisely weighed chemicals in alumina crucibles in air in an electric furnace (Vecstar, UK) equipped with SiC rods, maintaining a temperature of 1150 °C. The melts were stirred multiple times to achieve a satisfactory level of homogeneity. Once uniform, the melts were poured into preheated stainless steel molds of the specified dimensions. The resulting samples were promptly placed in an annealing muffle set to 350 °C to promote uniform temperature distribution that reduces the risk of cracking or warping, and enhances the overall stability and strength of the prepared glasses. Then, the muffle was turned off, allowing the glass samples to cool to room temperature at a controlled rate of 30 °C/hour. Figure 1 illustrates the prepared glasses before and after gamma irradiation.

**Table 1:** The chemical composition of the prepared glasses in Mol. %.

Sample	P_2_O_5_	B_2_O_3_	Na_2_O	Al_2_O_3_	CaF_2_	RE
Undoped	44	20	19	5	12	0
Ce -glass	44	20	19	4.8	12	0.2 CeO_2_
Ho-glass	44	20	19	4.8	12	0.2 Ho_2_O_3_
Sm-glass	44	20	19	4.8	12	0.2 Sm_2_O_3_

### Methods of characterization

The prepared glass samples were subjected to x-ray diffraction analysis to determine the likelihood of any crystalline phases forming during the preparation process. Fine-grained powder samples were analyzed using a diffractometer equipped with a nickel filter and a copper target. The measurements were conducted with a Philips PW-1390 X-ray diffractometer.

Optical absorption spectra were employed to comprehensively evaluate the optical characteristics of precision-polished glass specimens, encompassing both undoped variants and those incorporating rare-earth dopants. Measurements were executed with a JASCO V-570 spectrophotometer (Japan), systematically probing the wavelength range of 200–2500 nm to reveal key absorption features associated with electronic structures and dopant interactions within the glass network. All samples maintained a consistent thickness of ~ 2 mm (± 0.1 mm), which guaranteed reproducible optical path lengths and enhanced the reliability of quantitative spectral analysis.

Photoluminescence excitation and emission spectra were systematically recorded at room temperature to characterize the luminescent properties of the samples. For this purpose, a high-performance Jasco FP-6500 fluorescence spectrophotometer (manufactured in Japan) was employed, featuring a powerful xenon arc lamp as the primary excitation light source. This instrument setup enabled precise measurement of both excitation and emission profiles across relevant spectral ranges, providing critical insights into the electronic transitions and energy levels within the glass matrix under investigation.

The determination of chromaticity coordinates (x, y) played a pivotal role in evaluating the color characteristics exhibited by the synthesized glasses. These coordinates were computed following the methodology detailed in reference^16^, which characterizes emitted colors via the three standard color-matching functions: x̄(λ), ȳ(λ), and z̄(λ). For non-monochromatic light sources characterized by their spectral relative power distribution P(λ), the tristimulus values (X, Y, Z) are derived through integration of these functions weighted by the emission spectrum, enabling precise quantification of perceived color in the prepared glasses.

1 $$X=\int P\left(\lambda \right)\overline{x }\left(\lambda \right)d\lambda$$

2 $$Y=\int P\left(\lambda \right)\overline{y }\left(\lambda \right)d\lambda$$

3 $$Z=\int P\left(\lambda \right)\overline{z }\left(\lambda \right)d\lambda$$

The tristimulus values X, Y, and Z describe the intensities of the red, green, and blue primaries required to reproduce the color *P(λ )*. Based on these values, the chromaticity coordinates *x* and *y* are obtained using the relations provided in^17,18^.

4 $$x=\frac{X}{X+Y+Z}$$

5 $$y=\frac{Y}{X+Y+Z}$$

6 $$z=\frac{Z}{X+Y+Z}$$

The technique of the thermoelectricity measurements with their details were like that in previous work of reference^19^, the thermoelectric power, also known as the Seebeck coefficient (ϕ), was determined by placing the sample between two copper electrodes in a sandwich configuration^19^. To create a temperature gradient necessary for the measurement, a differential method was employed, which involved increasing the temperature of one surface of the sample using a small heating coil. This setup allowed for a controlled temperature difference across the sample, enabling accurate assessment of the thermoelectric properties. By measuring the resulting voltage generated due to this temperature gradient, we could effectively calculate the Seebeck coefficient. The thermoelectricity measurements (ϕ) were calculated by using the following relation^19:^

7 $$\phi\,=\,lim_{\triangle T \rightarrow 0}\left(\frac{\triangle V}{\triangle T}\right)$$

where (Δ*T*) is a slight as compared to the absolute temperature. From room temperature to 500 K, the thermoelectricity was measured over a broad temperature range.

The measurements of the electrical conductivity were like that in the articles by Kh. Roumaih^20,21^, for the prepared glass samples were conducted using well-polished specimens that had a rectangular shape, specifically with dimensions measuring 10 mm in length, 5 mm in width, and a thickness of 2 mm. This assessment was performed employing the two-probe method, which involved the application of a complex impedance technique to accurately evaluate the electrical properties of the materials. Fortunately, both DC and AC parameters can be measured simultaneously using the LCR meter (HIOKI IM3536). Where the calculation of the ***dc*** and ***ac*** parameters can be written as:

8 $$\sigma_{dc} = \frac{t}{{A \times R_{dc} }}$$

9 $$\sigma_{ac} = \frac{t}{A \times Z}$$

and

10 $$\varepsilon = \frac{(C \times t)}{A}$$

where σ is the conductivities, *A* is the surface area of the sample (m^2^), Z is the measurement of the impedance (in Ω units), and R_***dc***_ is the measurement of the ***dc*** resistance (in Ω units), ε is the dielectric constant, *C* is the measurement of the capacitance (in Farad), *t* is the thickness of the sample (m).

## Results and discussions

### X-ray diffraction (XRD) measurements

**Fig. 2:**
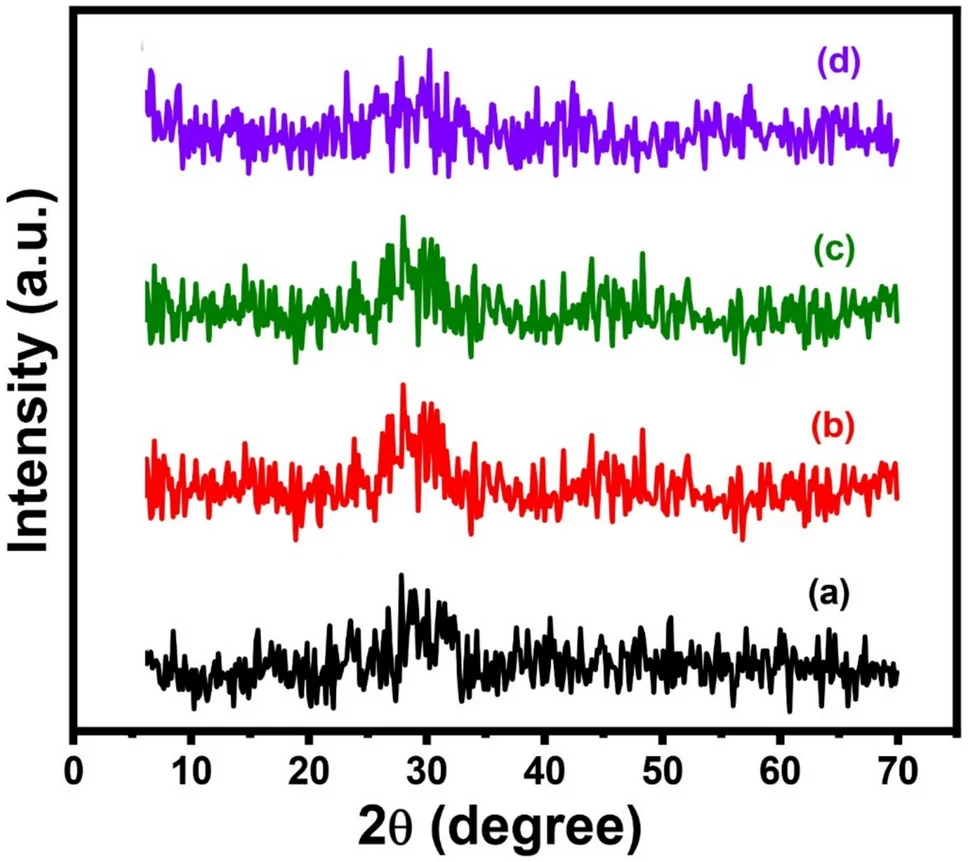
X-ray diffraction patterns of the prepared glasses where (**a**) undoped, (**b**) CeO_2_—, (**c**) Ho_2_O_3_—, (**d**) Dy_2_O_3_ – doped glasses.

Figure 2 of the X-ray diffraction analysis demonstrates the amorphous nature of the prepared glasses, highlighted by the absence of sharp diffraction peaks that are typical of crystalline structures. Instead, the XRD patterns reveal broad humps or halos, indicative of non-crystalline materials. This absence of distinct peaks confirms that the glasses exhibit a disordered atomic arrangement, solidifying their classification as amorphous.

### UV–Vis-NIR optical absorption measurements

**Fig. 3:**
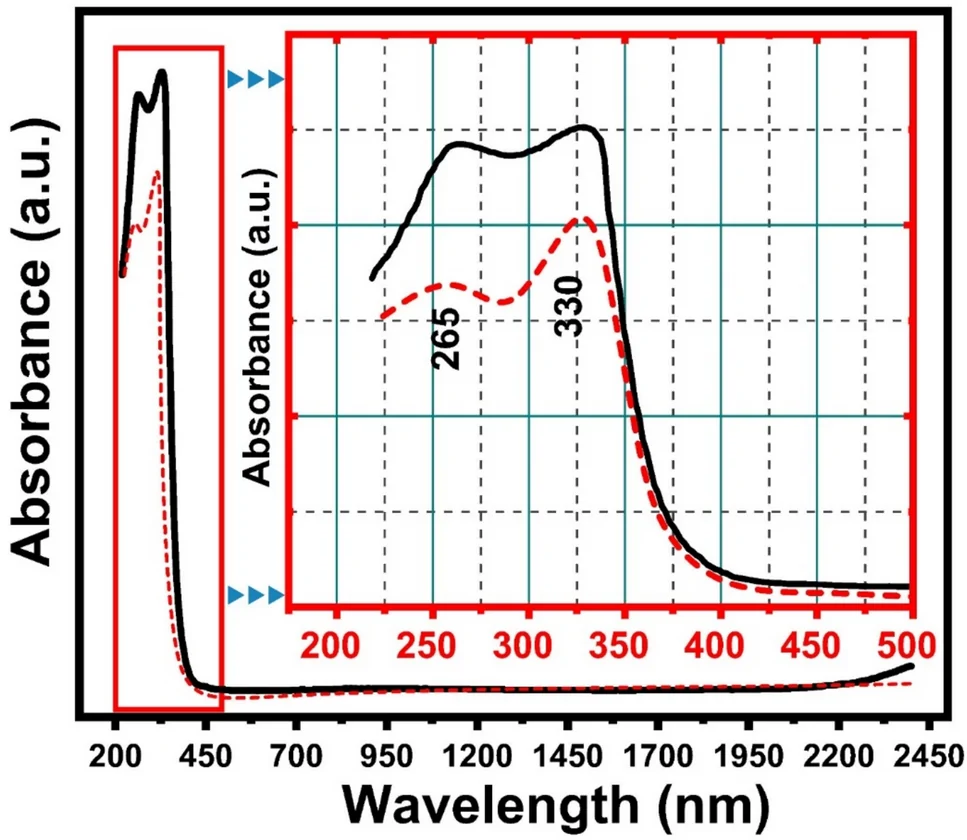
UV–VIS-NIR absorption spectra of the undoped glass before (black line) and after 75 KGy (red line) dose of gamma irradiation.

Figure 3 presents the ultraviolet–visible absorption spectra for the undoped base glass before gamma irradiation, compared directly with the spectrum obtained after exposure to a gamma dose of 75 kGy. In the unirradiated condition, the optical absorption profile exhibits a pronounced and broad absorption band within the UV region, characterized by two well-defined peaks centered at approximately 265 nm and 330 nm; notably, no additional absorption features are observed extending toward the longer wavelengths up to the limit of the measurement range.

The emergence of these characteristic UV absorption bands in the undoped glass can be attributed to the presence of trace iron impurities inherent in the raw chemical precursors, as originally proposed by Duffy^22^ and further substantiated through comprehensive investigations by Ehrt et al.^23,24^. Subsequent extensive research conducted by numerous glass scientists has consistently corroborated this mechanism, revealing analogous distinct UV absorption features in a variety of undoped borate and/or phosphate based glass systems^23–25^; consequently, the same explanatory framework centering on inadvertent iron contamination as the primary culprit for intrinsic UV absorbance has been widely embraced across the field, providing a robust interpretive lens for understanding baseline optical behavior in technically prepared silicate and borate glasses before any deliberate doping or irradiation treatments.

**Fig. 4:**
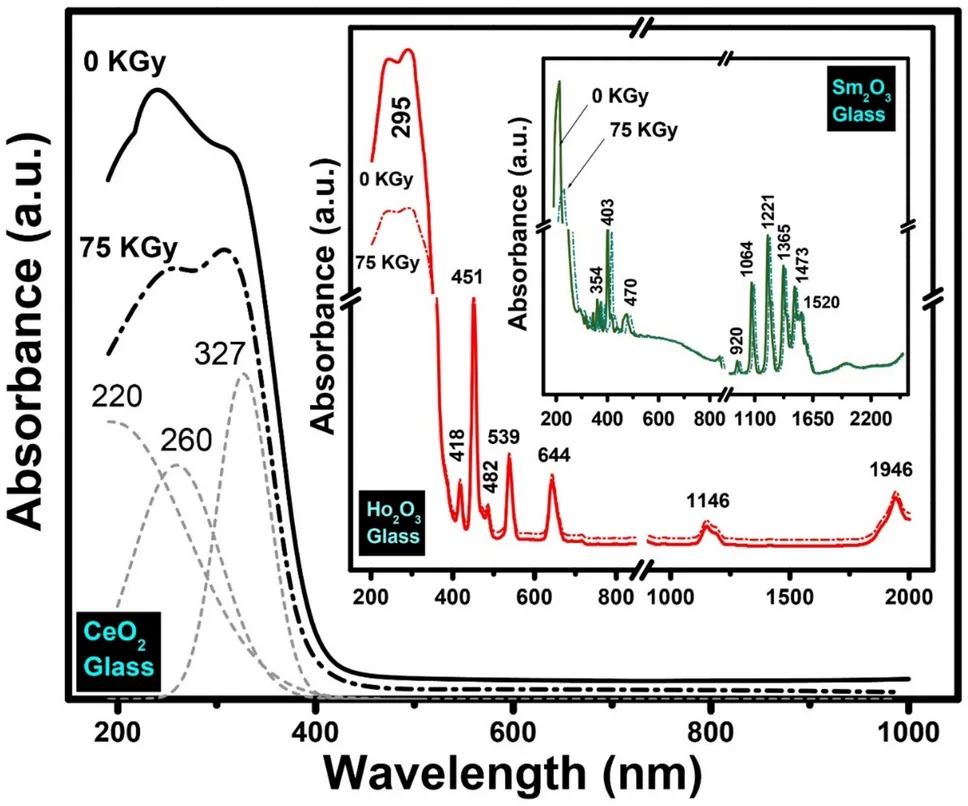
UV–VIS-NIR absorption spectra of the rare-earth oxides doped glass before and after 75 KGy dose of gamma irradiation.

Following the 75 kGy gamma irradiation, the spectrum of the doped glass maintains this characteristic strong UV absorption behavior, closely mirroring that of the unirradiated counterpart, albeit with a modest reduction in the peak intensities at both 265 nm and 330 nm, suggesting subtle modifications in the electronic structure or defect states induced by the radiation exposure without fundamentally altering the overall absorption profile. The UV–visible absorption spectra of the CeO_2_-doped glass samples recorded before and after gamma irradiation are presented in Fig. 4. The spectra are characterized by a strong and broad absorption peak in the ultraviolet region, consisting of three distinct absorption peaks centered at approximately 220, 260, and 327 nm. Throughout the entire measurement range, no additional absorption features are observed beyond these peaks. For samples that display a noticeable reduction in absorption intensity, the spectra obtained after gamma irradiation closely resemble those measured before irradiation, indicating that the overall spectral profile and peak positions remain essentially unchanged despite the decrease in intensity.

The behavior of ultraviolet absorption spectra of CeO_2_- doped glass can be understood from the optical characteristics of lanthanide ions, which generally produce sharp absorption features from the UV to near-infrared regions, although cerium and some related ions show relatively simple and weak spectra due to the stability of empty, half-filled, or filled 4f. shells^1^. Cerium exists in glass in two stable oxidation states, Ce^3+^ (4f^1^) and Ce^4+^(4f^0^), each contributing differently to the absorption spectrum^26–29^. The Ce^3^⁺ ions give rise to an intense and broad allowed 4f. → 5 d transition that typically appears in the UV region of oxide glasses, while Ce^4+^ absorption originates from an O^2-^ → Ce^4+^ charge-transfer transition occurring at relatively longer wavelengths. Previous studies^26–29^ have shown that Ce^4+^ exhibits a charge-transfer peak near 250 nm, whereas Ce^3+^ absorbs in the range of approximately 220—350 nm. Accordingly, the observed UV absorption in the present glasses is attributed to overlapping contributions from Ce^4^⁺ ions, which may interfere with UV bands associated with trace Fe^3+^ impurities, while the distinct band around 327 nm is assigned to the contribution of Ce^3+^ ions.

Figure 4 illustrates the absorption spectra of the synthesized Ho^3^⁺-doped glass before and after gamma irradiation across both the ultraviolet–visible (UV–Vis) and near-infrared (NIR) spectral regions. A prominent and intense absorption band appears in the UV region at approximately 295 nm, highlighting strong light-matter interactions at shorter wavelengths. Extending into the visible and NIR domains, the spectra exhibit seven well-defined absorption peaks located at roughly 418, 451, 482, 539, 644, 894, 1146, and 1946 nm. These peaks correspond to electronic transitions originating from the ground state ^5^I_8_ of the Ho^3+^ ions to various higher-lying excited states, specifically ^5^G_5_, ^5^G_6_, ^5^F_3_, ^5^F_4_, ^5^F_5_, ^5^I_6_, and ^5^I_7_, as corroborated by established references^30–32^. Among these, the transitions ^5^I_8_ → ^5^G_6_ (centered at 451 nm) and ^5^I_8_ → ^5^I_7_ (at 1946 nm) stand out as the most intense features within the visible and NIR regions, respectively, due to their enhanced oscillator strengths. Notably, the ^5^I_8_ → ^5^G_6_ transition demonstrates particular sensitivity to variations in the local ligand field inhomogeneity and the concentration of Ho^3+^ ions within the glass matrix, classifying it as a hypersensitive transition that adheres to the stringent selection rules of |ΔS|= 0, |ΔL|≤ 2, and |ΔJ|≤ 2^30–33^.

Figure 4 displays the ultraviolet–visible-near infrared absorption spectra of the glass sample doped with Sm_2_O_3_ before and after gamma irradiation, vividly illustrating the complex and nuanced mechanisms of light absorption exhibited by this material across an extensive wavelength spectrum. These spectral curves provide a thorough examination of the transformative effects that incorporating Sm_2_O_3_ has on the fundamental optical attributes of the host glass, including alterations in absorption coefficients, transmittance profiles, and overall photonic response, which are pivotal for understanding potential applications in luminescent devices, optical amplifiers, and radiation-resistant materials. Distinct absorption maxima are observed at roughly 354, 403, 470, 920, 1064, 1221, 1365, 1474, and 1520 nm, each attributable to specific intra-configurational f-f electronic transitions within the trivalent samarium ions (Sm^3+^) embedded in the amorphous glass network: namely, ^4^D_3/2_, ^6^P_3/2_, ^4^I_9/2_, ^6^F_11/2_, ^6^F_9/2_, ^6^F_7/2_, ^6^F_5/2_, ^6^F_3/2_, and ^6^F_1/2_, respectively^32,34–36^. This series of well-defined peaks not only reveals the characteristic sharp-line spectra typical of rare-earth dopants due to shielded 4f. electrons but also emphasizes the sophisticated dynamic coupling between the Sm^3^⁺ ions and the surrounding glass matrix, where local crystal-field perturbations split the excited-state manifolds, influencing peak positions, intensities, and bandwidths in ways that reflect the compositional and structural nuances of the host lattice.

### Optical bandgap and Urbagh energy

The optical bandgap (E_opt_) and Urbach energy (∆E) are important parameters for evaluating the optical and structural properties of rare-earth-doped glasses. The optical bandgap reflects the electronic structure and transparency of the glass, while the Urbach energy indicates the degree of structural disorder and defect states. Changes in these parameters due to rare-earth doping or gamma irradiation provide valuable insights into modifications of the glass network and their influence on the optical and luminescence performance.

The bandgaps were calculated from the optical absorption coefficient using the formula by Mott and Davis^37^ :

11 $$\alpha hv = B(hv - E_{opt} )^{n}$$

where; α is the absorption coefficient, *h* is Planck’s constant, *ν* is the frequency of the incident light, *E*_*opt*_​ is the optical bandgap energy, *B* is a constant that depends on the material, and *n* is a parameter that indicates the nature of the transition.

The Urbach energy (ΔE) was estimated from Urbach’s mathematical relationship (12)^38^ :

12 $$\alpha \left(\nu \right)=B exp\left(\frac{h\nu }{\Delta E}\right)$$

The ΔE values can be estimated by plotting drawn between lnα and photon of energy *hv* and taking the slope at the linear region of the curves. The estimated optical band gap and Urbach energy are given in Table 2.

**Table 2:** Optical band gap (Eopt) and urbach energy (∆E) of the prepared glass.

Sample code	Eopt (eV)		∆E (eV)	
	**0 KGy**	**75 KGy**	**0 KGy**	**75 KGy**
Undoped – glass	2.92	2.77	0.216	0.302
Ce- glass	2.73	2.63	0.220	0.269
Ho- glass	2.57	2.52	0.230	0.251
Sm-glass	3.55	3.43	0.420	0.467

The optical bandgap (Eopt) and Urbach energy (∆E) of the undoped and rare-earth-doped borophosphate glasses before and after 75 kGy gamma irradiation are summarized in Table 2. The results reveal that both rare-earth doping and gamma irradiation significantly influence the electronic structure and structural disorder of the glass network.

**Fig. 5:**
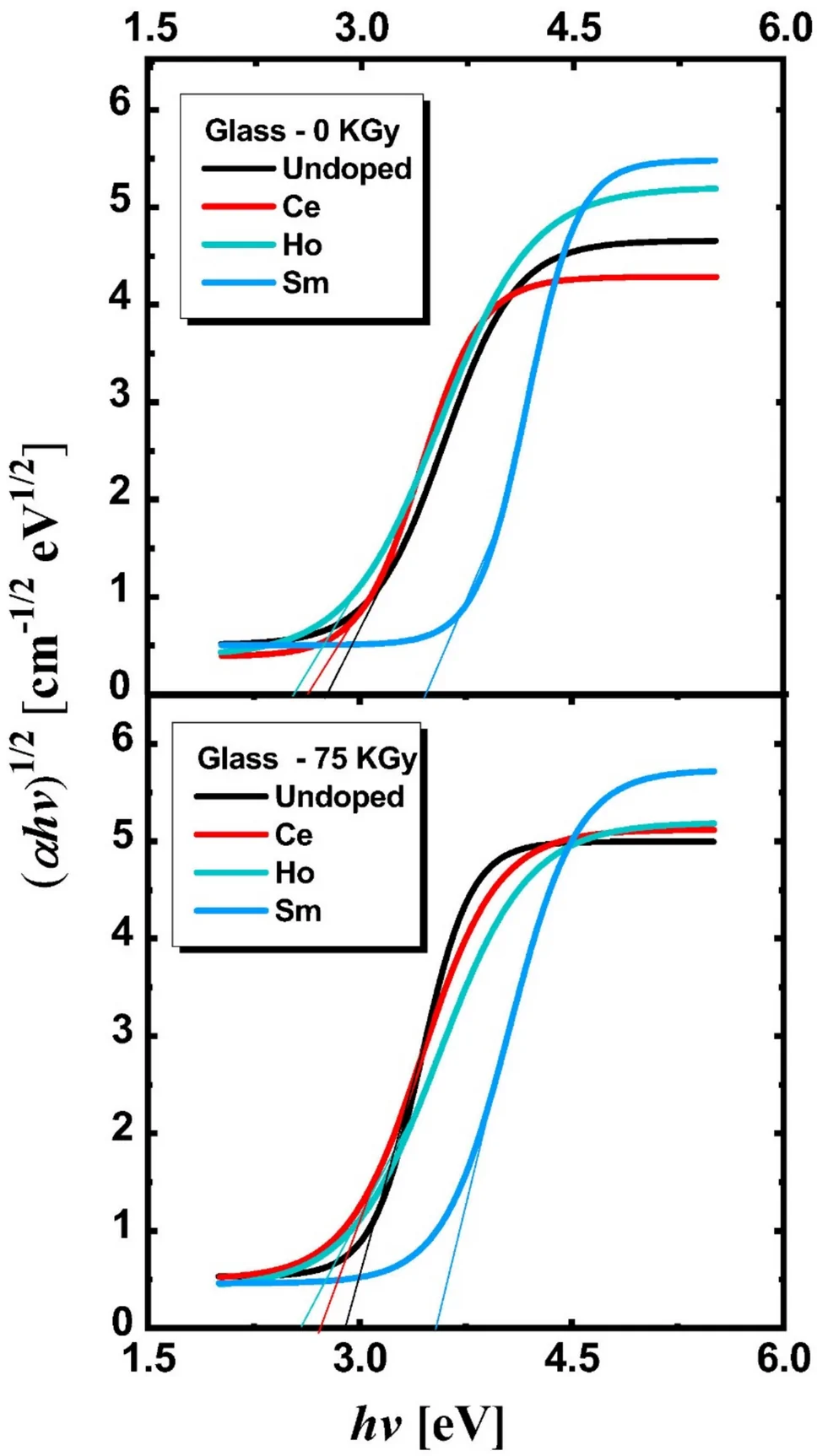
Optical bandgap of the prepared glasses before and after gamma irradiation.

According to Fig. 5, before irradiation, the undoped glass exhibits an optical bandgap of 2.92 eV, which decreases to 2.73 and 2.57 eV after doping with Ce^3^⁺ and Ho^3^⁺, respectively. The reduction in E_opt_ is attributed to the introduction of localized electronic states within the forbidden bandgap due to the incorporation of rare-earth ions and the formation of non-bridging oxygen (NBO) sites^39^. These defect states facilitate electronic transitions at lower photon energies, thereby narrowing the optical bandgap. In contrast, the Sm^3+^-doped glass shows the highest optical bandgap 3.55 eV, suggesting that Sm^3+^ promotes a relatively more compact and stable glass network with fewer localized defect states^39–43^. Following exposure to 75 kGy gamma irradiation, all glass samples exhibit a further decrease in the optical bandgap. The undoped glass shows the largest reduction from 2.92 to 2.77 eV, while the Ce^3^⁺-, Ho^3^⁺-, and Sm^3^⁺-doped glasses decrease to 2.63, 2.52, and 3.43 eV, respectively. This behavior is attributed to radiation-induced defect formation, including oxygen vacancies, trapped electrons, hole centers, and color centers, which generate additional localized states within the bandgap^39–44^. These newly formed defect levels reduce the energy required for electronic excitation, leading to bandgap narrowing after irradiation. The Urbach energy exhibits the opposite trend, increasing after gamma irradiation for all samples. The undoped glass shows an increase from 0.216 to 0.302 eV, while the Ce^3+^-, Ho^3+^-, and Sm^3+^-doped glasses increase from 0.220 to 0.269 eV, 0.230 to 0.251 eV, and 0.420 to 0.467 eV, respectively. The increase in Urbach energy indicates a greater degree of structural disorder and a higher density of localized states in the vicinity of the band edges. Gamma irradiation disrupts the borophosphate glass network by breaking B-O-B and P-O-P linkages and generating non-bridging oxygen atoms, thereby broadening the exponential absorption tail^39,44^.

Among the investigated compositions, the Ho^3+^-doped glass exhibits the smallest irradiation-induced changes in both optical bandgap and Urbach energy, suggesting relatively better resistance to radiation-induced structural disorder. Conversely, the undoped glass experiences the largest increase in disorder after irradiation, while the Sm^3+^-doped glass maintains the highest optical bandgap despite possessing the largest Urbach energy, indicating that the Sm^3+^ ions strongly influence both the electronic structure and the localized disorder of the glass network.

### Photoluminescence measurements

**Fig. 6:**
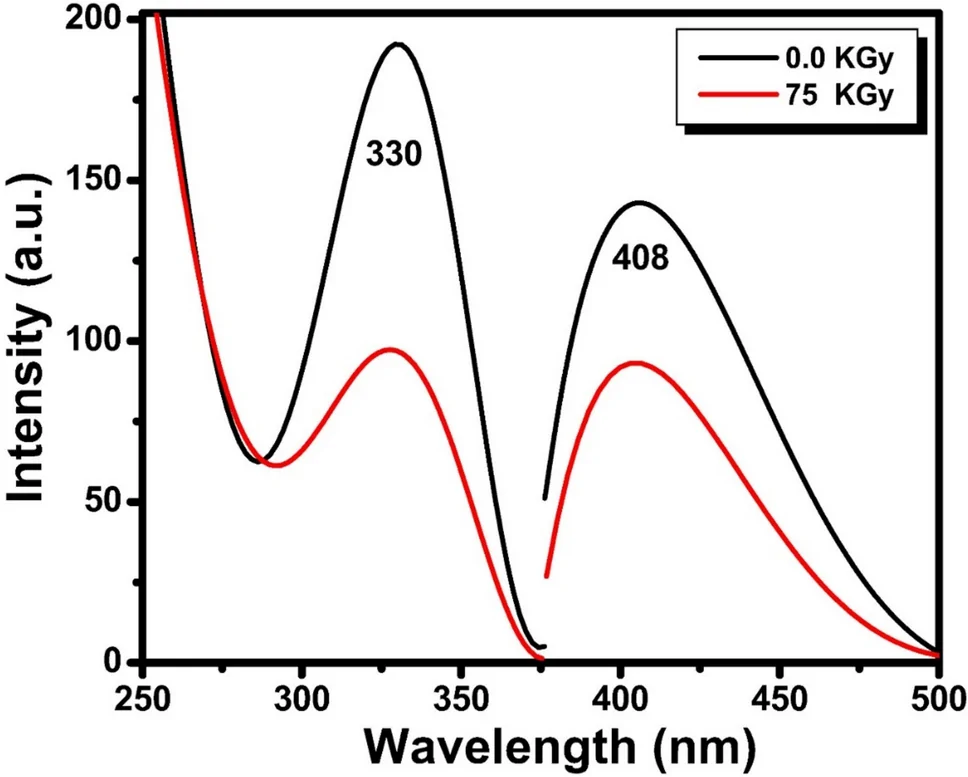
PL spectra of CeO_2_ – doped glass before and after gamma irradiation.

The photoluminescence spectra of cerium dioxide (CeO_2_) doped glasses, as depicted in Fig. 6, provide a detailed comparison of the optical behavior of these materials prior to and following exposure to gamma irradiation. Initially, the spectra recorded before irradiation clearly show a prominent and sharply intense excitation peak, which is centered approximately at a wavelength of 330 nm. Alongside this, there is a moderately broad emission band that appears around 408 nm. These spectral features represent the intrinsic luminescent properties of the CeO_2_-doped glass matrix under unaltered conditions. When the glasses are subjected to gamma irradiation, notable changes occur in the intensity of the excitation and emission spectra, although the positions of the peaks themselves remain largely unchanged. Specifically, the intensity of the excitation peak demonstrates a distinct decrease compared to the pre-irradiation state, indicating that the gamma rays induce alterations in the electronic or defect structure of the doped glass that impact its ability to absorb and re-emit light. In tandem with the excitation behavior, the emission spectra also similarly respond to gamma treatment; the emission band after irradiation exhibits a comparable decrease in intensity but retains its original spectral position around 408 nm. This parallel behavior between excitation and emission spectra under gamma irradiation implies that while the radiation exposure diminishes the luminescence efficiency, it does not significantly shift the energy levels of the luminescent centers within the doped glasses.

Previous studies^45,46^ have widely attributed the observed excitation spectrum to the presence of Ce^3^⁺ ions, interpreting it as arising from characteristic electronic transitions within these ions, specifically, the promotion of electrons from the excited 4f. orbital state to the higher-energy 5 d ground state configuration. This assignment makes intuitive sense given the well-documented luminescent properties of cerium in such doped glass systems, where the parity-allowed 5d–4f. transitions typically produce broad excitation bands in the near-UV region, often peaking around 300–350 nm depending on the host matrix and local coordination environment. The emission spectrum frequently displays a prominent peak at around 408 nm, stemming from the intrinsic fluorescence properties of cerium oxide within the amorphous host matrix. This blue-violet emission arises primarily from defect-related recombination processes involving oxygen vacancies and charge transfer transitions between Ce^4^⁺ and Ce^3^⁺ states.

The reduction in emission intensity observed in CeO_2_-doped glass following gamma irradiation exposure typically stems from radiation-induced structural disruptions and electronic modifications within the glass matrix, which quench luminescent centers and hinder efficient radiative recombination^47,48^. Gamma rays generate high-energy electrons that trigger processes like radiolysis of the glass network, creating oxygen vacancies, non-bridging oxygens (NBOs), and defect traps that compete with cerium-related emission pathways, such as the characteristic 5d-4f. transitions of Ce^3+^ or charge transfer involving Ce^4+^/Ce^3+^ pairs. This leads to increased non-radiative decay channels, where trapped charges dissipate energy as heat rather than light, resulting in diminished photoluminescence intensity at peaks like 408 nm, particularly at higher doses where crystallinity or local coordination around Ce ions degrades^45–48^.

**Fig. 7:**
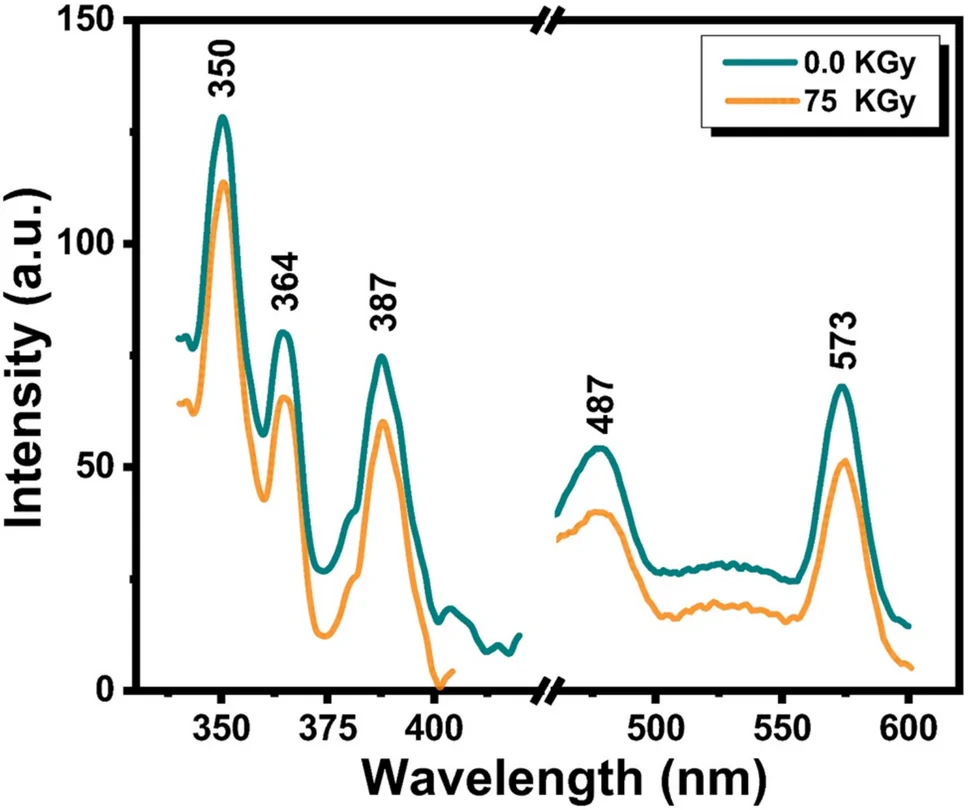
PL spectra of Ho_2_O_3_ – doped glass before and after gamma irradiation.

Figure 7 represents the photoluminescence spectra for Ho_2_O_3_—doped glass. In the excitation spectra, the glass exhibits three prominent peaks that are observed at wavelengths of 350, 364, and 387 nm. The intensity of these excitation peaks reveals a characteristic decrease after exposure to a 75 kGy dose of gamma irradiation. Turning to the emission spectra, these display two primary peaks centered at 487 nm and 573 nm. Notably, the intensities of the emission peaks also show a characteristic decrease after gamma irradiation.

The excitation spectral peaks observed at 350, 364, and 387 nm in the Ho^3^⁺-doped glasses are confidently attributed to the characteristic f-f electronic transitions originating from the ground state ^5^I_8_ of the Ho^3^⁺ ions to the higher-lying excited states ^5^G_3_, ^5^H_6_, and ^5^G_4_, respectively^49–51^. These assignments are based on well-established energy level diagrams for trivalent holmium ions in oxide glass matrices. Complementing these excitation features, the prominent emission peaks recorded at 487 nm and 573 nm are assigned to the radiative relaxation pathways ^5^F_3_ → ^5^I_8_ and ^5^F_4_ + ^5^S_2_ → ^5^I_8_, respectively, representing efficient luminescent decay channels from the blue-green and yellow emission manifolds back to the ground state^49–53^. These assignments not only reflect the population dynamics under the applied excitation conditions but also highlight the multi-phonon relaxation processes that populate the ^5^F₄/^5^S₂ levels from higher-lying states, a common phenomenon in Ho^3^⁺-doped amorphous materials. The consistency of these peak positions and relative intensities with extensive literature precedents spanning studies on Ho^3^⁺-doped silicate, phosphate, borate, and fluoride glasses serves as robust validation of the spectroscopic interpretations, while also underscoring the reproducibility of holmium’s luminescent signature across diverse host lattices.

**Fig. 8:**
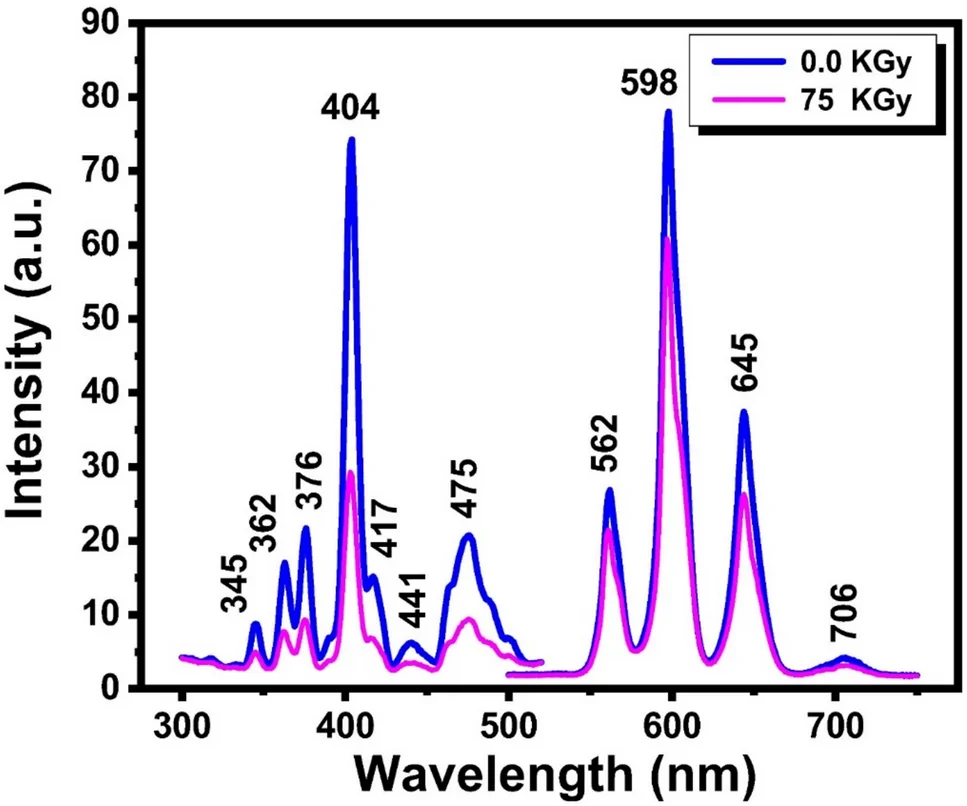
PL spectra of Sm_2_O_3_ – doped glass before and after gamma irradiation.

Figure 8 presents a comprehensive visualization of the photoluminescence spectra recorded for the Sm_2_O_3_-doped glass sample before and after exposure to gamma irradiation. In the excitation spectra, obtained under an excitation wavelength of λ_ex_ = 598 nm, a series of seven distinct peaks emerges prominently at wavelengths of 345, 362, 376, 404, 417, 441, and 475 nm. Among these, the peak at 404 nm exhibits the most intense signal, indicating it as the optimal excitation wavelength for the Sm^3+^ ions incorporated within the glass matrix. Notably, upon subjecting the sample to gamma irradiation, the intensities of all these excitation peaks experience a measurable reduction. This diminution in peak heights suggests a disruption in the electronic structure of the Sm^3+^ dopant ions, potentially arising from radiation-induced defects, such as non-bridging oxygen hole centers or other trap states that quench the excitation efficiency^54^.

Complementing these observations, the emission spectrum excited at λ_ex_ = 404 nm displays four well-defined characteristic emission peaks, located approximately at 562, 598, 645, and 706 nm. These peaks, which are hallmarks of trivalent samarium (Sm^3+^) ions in amorphous hosts like glass, also undergo a consistent decrease in their emission intensities following gamma ray exposure. The reduction in luminescence output points to a degradation in the radiative recombination pathways, where gamma-induced structural modifications in the glass network, possibly including bond breaking, ionization, or the formation of color centers, compete with the desired luminescent transitions, thereby suppressing the overall emission yield. The origins of these emission bands can be precisely attributed to electronic transitions within the 4f^5^ configuration of the Sm^3^⁺ ions. Specifically, the band at 562 nm corresponds to the hypersensitive transition from the excited state ^4^G₅/₂ to the ground state ^6^H_5/2_ (^4^G_5/2_ → ^6^H_5/2_). The prominent orange emission at 598 nm arises from ^4^G_5/2_ → ^6^H_7/2_, while the red emission at 645 nm is linked to ^4^G_5/2_ → ^6^H_9/2_. Finally, the band at 706 nm in the near-infrared region stems from the transition ^4^G_5/2_ → ^6^H_11/2_ (noting a likely typographical correction from ^6^H_13/2_ in the original for standard notation consistency, as per literature)^55–59^. These assignments align seamlessly with extensive reports in the literature by various research groups, who have similarly identified these transitions in Sm^3+^-doped glasses and confirmed their sensitivity to host matrix perturbations and external stimuli like ionizing radiation.

**Fig. 9:**
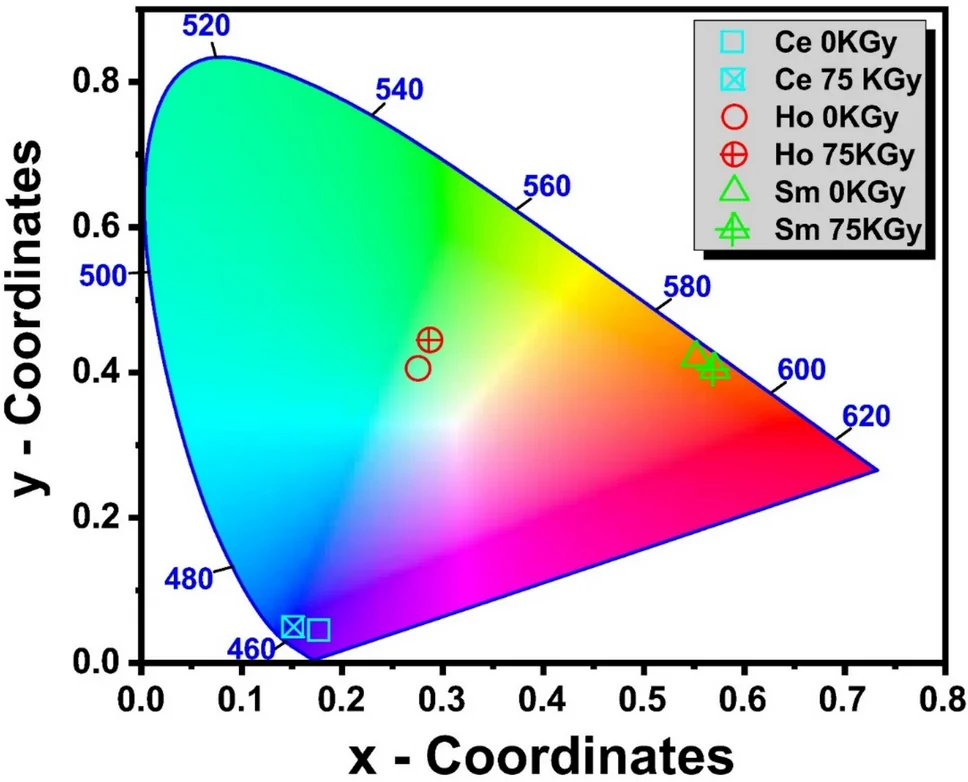
The CIE chromaticity coordinates of the undoped and rare earth – doped glass before and after gamma irradiation.

Figure 9 illustrates the CIE 1931 chromaticity diagram, a foundational color space model developed by the International Commission on Illumination in 1931 that maps human color perception onto a two-dimensional plane using x and y coordinates derived from tristimulus values, thereby enabling precise visualization and quantification of the color gamut produced by photoluminescence (PL) emissions from the various doped glass samples under systematic investigation in this study. The values of the CIE coordinates are listed in Table 3. Specifically, for the CeO_2_—doped glass composition, the chromaticity coordinates register at (0.177, 0.045) in the pristine, unirradiated condition reflecting the characteristic emission peak typically associated with cerium ions transitioning from the 5 d excited state to the 4f. ground state and undergo a subtle yet measurable shift to (0.151, 0.049) upon subjection to controlled gamma irradiation, with both loci remaining steadfastly anchored within the violet region of the diagram, a domain characterized by low y-values and moderate x-values that correspond to bluish-purple hues perceptible to the human eye under typical viewing conditions, thereby suggesting that the radiation-induced defects, such as oxygen vacancies or cerium valence state alterations from Ce^3^⁺ to Ce^4^⁺^26,27^, exert only marginal influence on the overall emission centroid without precipitating a wholesale relocation across color boundaries.

**Table 3:** CIE chromaticity coordinates of the prepared rare-earth doped – glass.

Sample code	Before irradiation		After irradiation	
	**x-coordinate**	**y-coordinate**	**x-coordinate**	**y-coordinate**
**Ce-glass**	0.177	0.045	0.151	0.049
**Ho-glass**	0.275	0.405	0.287	0.444
**Sm-glass**	0.552	0.420	0.570	0.404

In marked contrast, the Ho_2_O_3_-doped glass manifests pre-irradiation coordinates of (0.275, 0.405), emblematic of the sharp-line green emissions arising from the hypersensitive ^5^F_3_ → ^5^I_8_ transition of Ho^3+^ ions amidst the glassy host matrix, which evolve modestly to (0.287, 0.444) in the post-irradiation scenario, with these points consistently and robustly ensconced within the verdant green sector of the CIE space a region defined by intermediate x-values around 0.2–0.4 and elevated y-values indicative of wavelengths peaking near 540–550 nm thus underscoring an exemplary degree of color stability and spectral resilience under gamma ray bombardment, a trait attributable to the shielded nature of 4f.−4f electric dipole-forbidden transitions that are comparatively impervious to host lattice perturbations induced by high-energy photons^26–28^. Complementing these observations, the Sm_2_O_3_—doped glass sample delineates chromaticity coordinates of (0.552, 0.427) prior to irradiation, capturing the warm, intense reddish-orange luminescence governed by the intra-configurational f-f transitions of Sm^3+^ ions such as ^4^G_5/2_ → ^6^H_7/2_ and ^4^G_5/2_ → ^6^H_9/2_ that dominate the visible spectrum around 600–650 nm, shifting incrementally to (0.570, 0.404) following the irradiation protocol, wherein both positions steadfastly occupy the reddish-orange expanse of the diagram, characterized by high x-values exceeding 0.5 and moderate y-values that evoke saturated amber-to-crimson tonalities, thereby accentuating the dopant-specific luminescent signatures and the nuanced interplay between rare-earth ion electronic structures, amorphous silica-based host environments, and cumulative radiation damage effects that subtly modulate emission intensities without compromising the perceptual color identity essential for applications in radiation dosimetry, solid-state lighting phosphors, or scintillator materials deployed in harsh radiative milieus.

Regarding the previous optical behavior of the prepared glasses, the decrease in absorption intensity after irradiation is generally attributed to the formation of irradiation-induced defects and structural disorder, which modify the electronic structure of the material. High-energy irradiation (γ-rays) generates point defects such as vacancies, interstitials defects, and dangling bonds that act as trapping centers for charge carriers. These defects can reduce the density of optically active absorbing centers, alter the local bonding environment, and decrease the oscillator strength of electronic transitions. In addition, irradiation may induce partial amorphization, lattice distortion, or chemical bond breaking, leading to a reduction in the probability of optical transitions and, consequently, a decrease in the absorption intensity^40,41^. In addition, irradiation may induce lattice distortion, partial amorphization, or degradation of luminescent centers, which further reduces the radiative recombination efficiency and photoluminescence quantum yield. As the concentration of irradiation-induced defects increases with dose, the density of non-radiative recombination centers also increases, leading to progressive quenching of the luminescence intensity^40,41^.

### Radiation-induced luminescence quenching mechanism

Gamma irradiation induces photoluminescence quenching in Ce^3^⁺, Ho^3^⁺, and Sm^3^⁺ co-doped borophosphate glasses through the formation of radiation-induced defects within the glass network. High-energy gamma photons generate oxygen vacancies, non-bridging oxygen sites, trapped electrons, and color centers, which act as non-radiative recombination centers (as represented in Fig. 1). These defects compete with the radiative transitions of the rare-earth ions by providing additional pathways for non-radiative energy dissipation, thereby reducing the PL intensity. Furthermore, gamma irradiation may partially oxidize Ce^3+^ to Ce^4+^, decreasing the number of optically active Ce^3+^ sensitizer ions and reducing the energy transfer efficiency from Ce^3+^ to Ho^3+^ and Sm^3+^. Structural modifications of the borophosphate glass network also alter the local environment around the rare-earth ions, further enhancing non-radiative relaxation. Although the emission intensities decrease with increasing irradiation dose, the characteristic emission peaks remain nearly unchanged because the 4f. electronic states of Ho^3+^ and Sm^3+^ are effectively shielded from the surrounding matrix. Thus, the observed PL quenching is mainly attributed to defect-assisted non-radiative relaxation, reduced Ce^3^⁺ sensitization, and radiation-induced structural disorder rather than degradation of the rare-earth luminescent centers^60^.

### Electrical properties

#### Seebeck coefficient (ϕ)

**Fig. 10:**
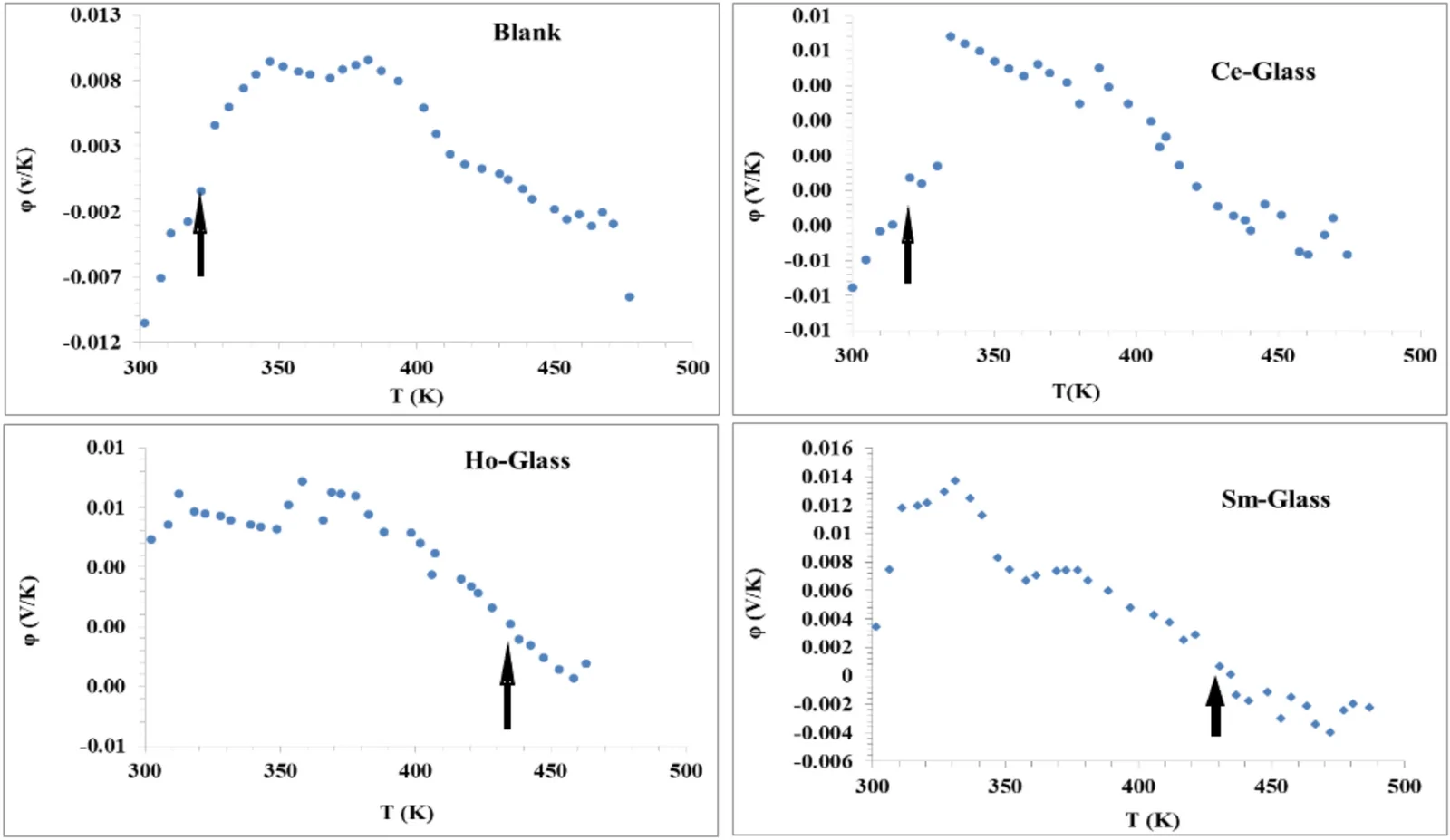
Thermoelectric power of blank and rare – earth doped glass.

Figure 10 shows the temperature-dependent measurement of the Seebeck coefficient φ (T) from near room temperature up to T = 500 K. We see that, the coefficient’s non-linear temperature dependence reflects each material’s unique conductivity mechanism. The Seebeck coefficient exhibits a nonlinear relationship for all samples, suggesting that each is governed by distinct conduction mechanisms characteristic of its composition. In detail, the blank sample exhibits charge carrier behavior similar to that of the cerium sample, although their transition temperatures (Ts) differ slightly, where Ts for blank is 374 K but for cerium doped is 335 K. Both samples initially possess negative charge carriers, which transition to positive charge carriers (As the arrow indicates) to reach the max positive value of the Seebeck coefficient φ at Ts, and then decrease to reach negative value. In contrast, the samarium and holmium samples start with positive charge carriers and switch to negative at approximately 430 K and 435 K, respectively. The transition temperatures (Ts) of samarium and holmium samples are 331 and 358 K, respectively.

#### DC conductivity

**Fig. 11:**
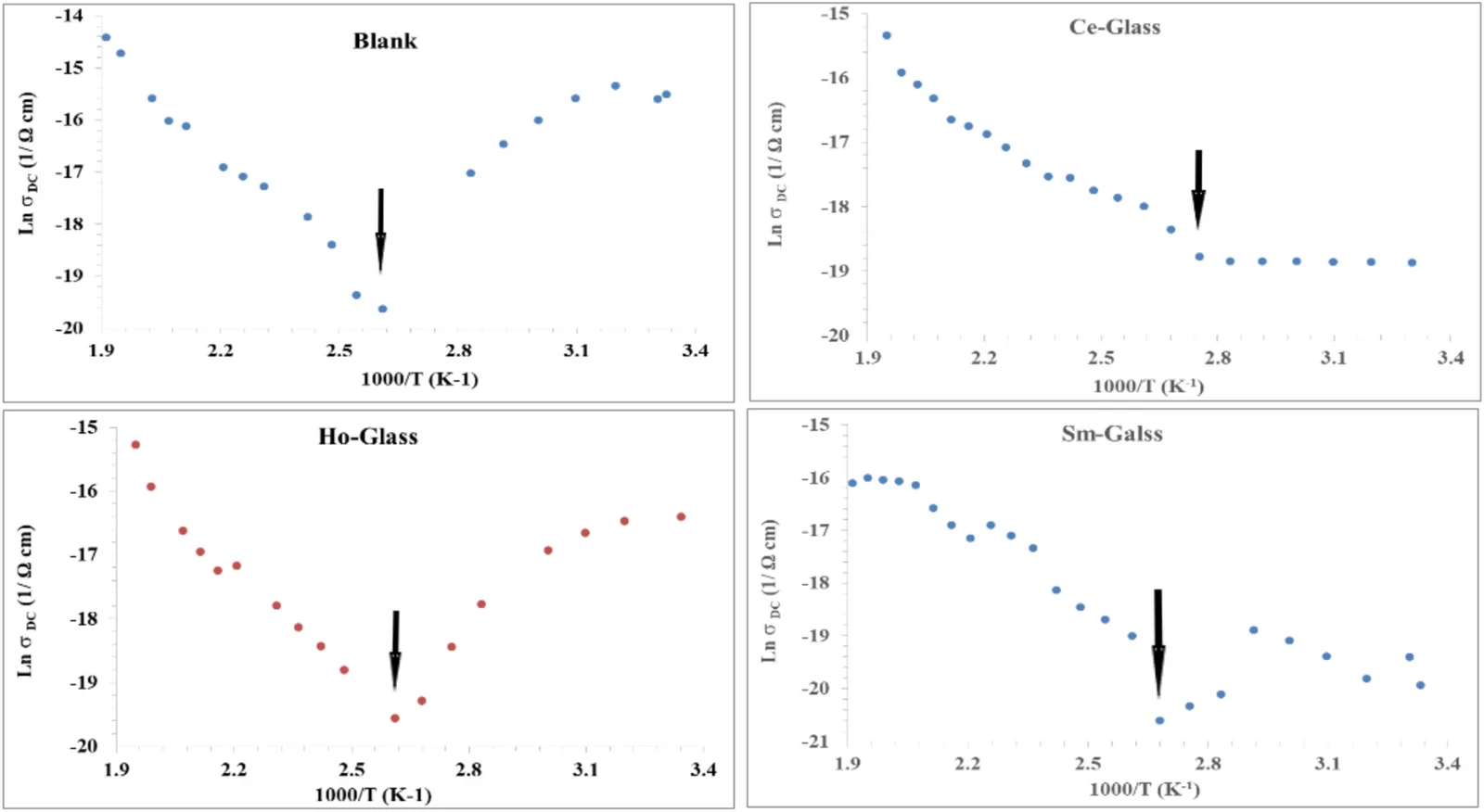
DC conductivity of blank and rare – earths doped glass before gamma irradiation.

Figure 11 presents the DC electrical conductivity of blank and rare-earth-doped (CeO_2_, Ho_2_O_3_, Sm_2_O_3_) glass samples as a function of temperature before gamma irradiation, revealing a characteristic non-monotonic behavior observed across all compositions: conductivity decreases progressively with increasing temperature up to a minimum at approximately 383 K for blank and Ho-doped, but for Sm-doped at 373 K, and for Ce-doped at 363 K, followed by an increase with temperatures. This initial decline in the low-temperature regime arises from enhanced carrier localization and trapping at defect sites or structural rearrangements within the amorphous glass network, where thermal activation promotes phonon-assisted scattering that impedes charge transport, a phenomenon consistent with thermoelectric power measurements and with other studies of vanadium-phosphate and alkali-containing oxide glasses^24,61,62^. In contrast, the upturn reflects a transition to thermally activated hopping conduction, where sufficient energy overcomes trapping barriers, allowing delocalization of charge carriers and modest conductivity enhancement governed by activation energies typically in the 0.3–0.4 eV range, mirroring behaviors in Li_2_O-doped glasses^24,61^. The persistence of a dual-regime pattern in rare-earth-doped samples indicates that dopants primarily modify local network rigidity and non-bridging oxygen sites without fundamentally altering the intrinsic conduction mechanism, underscoring the role of glass composition in balancing electronic and ionic contributions to DC transport.​

**Fig. 12:**
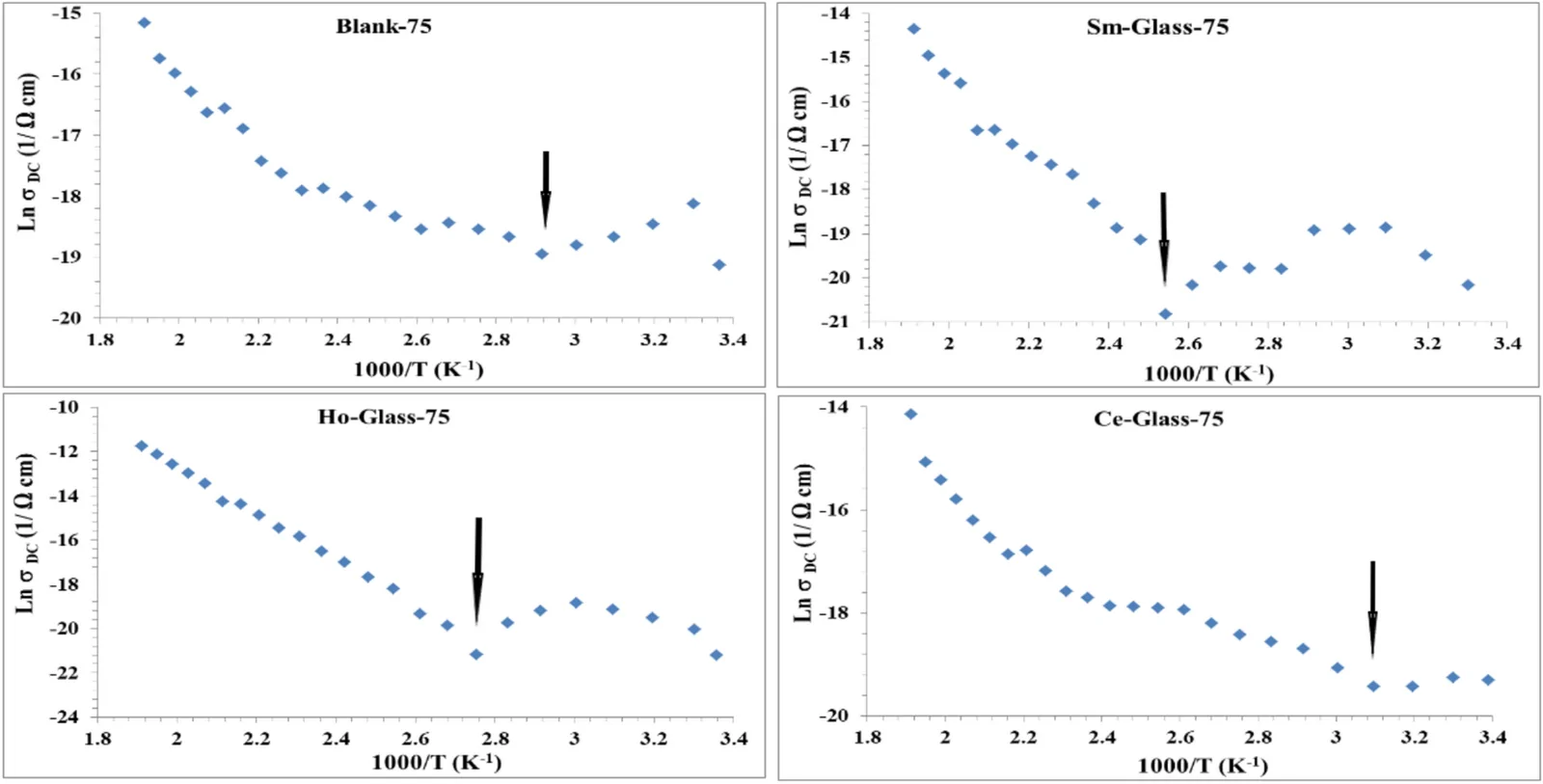
*Dc* conductivity as Ln(σ_*dc*_) versus 1000/T for blank and rare – earths doped glass after gamma irradiation.

Figure 12 depicts the fluctuation of *dc* conductivity (ln σ_***dc***_) with temperature (1000/T) for both the blank glass and those doped with rare-earth oxides, following exposure to a gamma irradiation dose of 75 kGy. The figure clearly a decrease in transition temperatures compared with the corresponding values before irradiation (see Table 4, except for Sm-doped). The decreases of Ts_DC_ can be attributed to irradiation-induced modifications in the glass structure, including the generation of defects, increased network densification or compaction, and the formation of localized trapping centers^63–65^. Such radiation-related defects increase the activation energy required for charge transport and significantly hinder the mobility of charge carriers by promoting enhanced scattering, localization, and recombination processes within the amorphous glass matrix. Although the Ts_DC_ values are markedly decreased after irradiation, the characteristic non-monotonic temperature dependence of DC conductivity remains evident. Specifically, the conductivity initially decreases with increasing temperature up to Ts_DC,_ followed by an increase with temperature. This behavior suggests that gamma irradiation intensifies lattice disorder and phonon-assisted interactions across the entire temperature range, thereby suppressing both electronic hopping and ionic conduction mechanisms. These observations are in good agreement with previously reported studies on irradiated borate and oxide glass systems, where similar gamma doses on the order of 75 kGy resulted in notable reductions in conductivity^55–57^. Moreover, the presence of rare-earth dopants does not appear to counteract the detrimental effects of irradiation, indicating that although CeO_2_, Ho_2_O_3_, and Sm_2_O_3_ alter the local glass structure before exposure, radiation-induced damage becomes the dominant factor governing electrical transport after irradiation.

**Table 4:** The values of DC activation energy (eV) for all glasses samples before and after gamma irradiation.

**Sample**	Ts_DC_ (K)	E1	E2	E3	Ts_DC_(K)	E1	E2
	**samples before gamma irradiation**				**after gamma irradiation**		
Blank	383	0.0118	−0.0121	–	343	0.0400	−0.0226
Ho-glass	383	0.0174	−0.0137	–	363	−0.0080	–
Sm-galss	373	−0.0318	0.0106	−0.0145	393	−0.0131	–
Ce-galss	363	−0.4203	−0.0212	–	323	−0.0213	–

Generally, the temperature-dependent electrical conductivity can be expressed as^20,21^:

13 $$\sigma_{dc} = \sigma_{o} \times \exp \left( {\frac{{ - E_{a} }}{{K_{B} T}}} \right)$$

where *σ*_o_ is the conductivity at zero temperature, K_B_ is the Boltzmann constant, and *E*_*a*_ is the activation energy for charge conduction. Equation (12) can be expressed as:

14 $$Ln\sigma_{dc} = \left( {\frac{{ - E_{a} }}{{K_{B} T}}} \right) + const.$$

The variation in the conductivity with temperature showed (Fig. 12) several slopes with different signs, i.e., for each region, there is a slope. The activation energies are tabulated in Table 4. The negative value of the activation energy, in some range of temperature, is due to the conductivity decrease with increasing temperature i.e. these samples, may be have conductor behavior in this range of temperature. The observed negative activation energy indicates that the electrical conductivity decreases with increasing temperature over the investigated temperature range, corresponding to a negative temperature coefficient of conductivity (NTCC). This behavior does not necessarily imply metallic conduction; rather, it may arise from changes in the dominant charge transport mechanism, such as carrier trapping and de-trapping, defect-assisted hopping, or structural relaxation within the glass network^37,42,66^. In rare-earth-doped oxide glasses, gamma irradiation and the presence of localized defect states can modify the distribution of charge carriers and hopping pathways, leading to anomalous temperature-dependent conductivity. Consequently, the apparent negative activation energy reflects the increasing influence of these competing non-thermally activated processes rather than a true thermally activated conduction mechanism^37,42,66^. Similar observations have been reported in disordered oxide glasses and amorphous semiconductors, where transitions between different conduction mechanisms produce negative apparent activation energies over limited temperature intervals. Therefore, the negative activation energy should be interpreted as evidence of a change in the dominant conduction process rather than as an indication of metallic behavior^37,42,66^.

#### AC conductivity

**Fig. 13:**
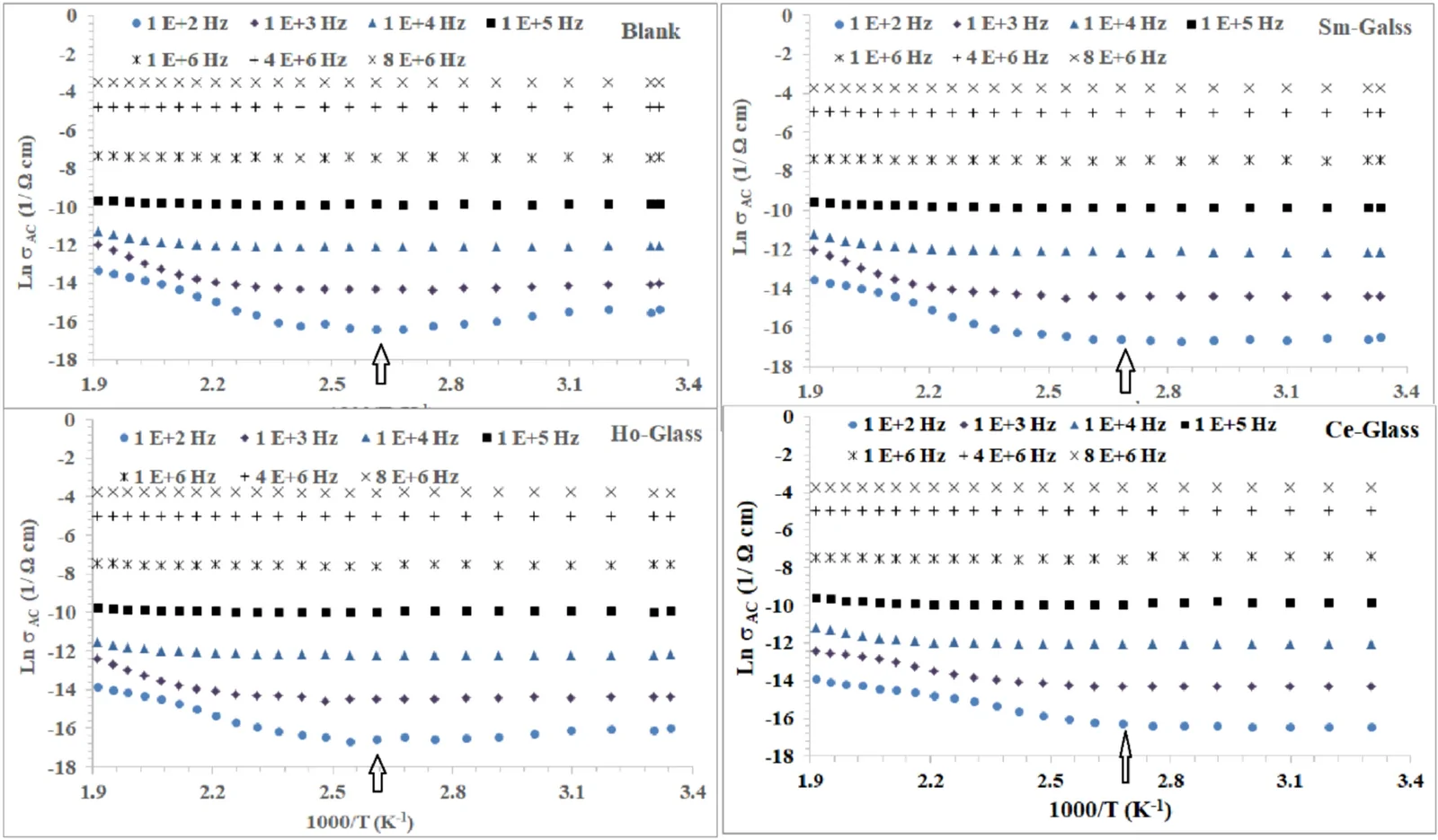
AC conductivity of blank and rare – earths doped glass with 1000/T at different frequencies before gamma irradiation.

**Fig. 14:**
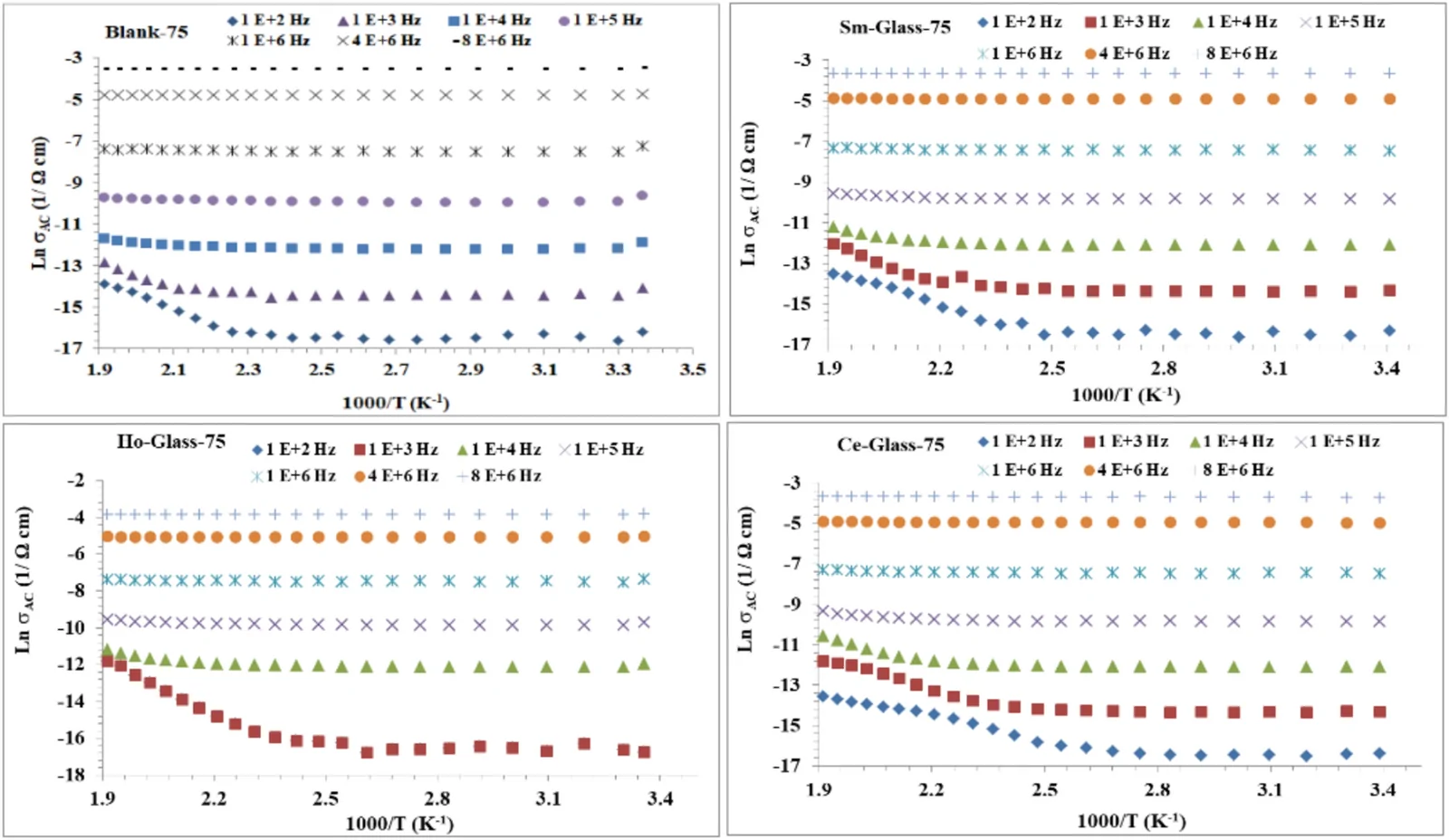
AC conductivity of blank and rare – earths doped glass with 1000/T at different frequencies after gamma irradiation.

Figures 13 and 14 present a comprehensive illustration of how the AC conductivity varies as a function of temperature at different frequencies for all glass samples under investigation. In all samples, both before and after irradiation, the AC conductivity exhibits the same behavior, i.e., remaining constant with temperature variations for all frequency ranges, except the range of 100–100,000 Hz. In the low-frequency range (100–100,000 Hz), the AC conductivity remains constant up to approximately the transition temperature Ts_DC_, after which it begins to increase. This means that the variation in AC conductivity with temperature is independent of the temperature. Furthermore, doping rare-earth oxides does not affect the behavior of AC conductivity, nor is it influenced by the gamma irradiation dose of 75 KGy. This behavior may be attributed to the mechanism of conductivity in these glasses, which involves ionic conduction from the glass material^67^. The presence of Ce^3^⁺/Ce^4^⁺, Ho^3^⁺, and Sm^3^⁺ ions is believed to disrupt the glass matrix, reduce potential barriers, and enhance the mobility of charge carriers, thereby leading changes in the values of AC conductivity^64,68,69^.

In all investigated glass samples, the AC conductivity increases with rising temperature, a phenomenon consistently observed across all compositions. This conductivity behavior fundamentally arises from the enhanced mobility of charge carriers within the amorphous glass matrix, facilitated by thermal activation processes^64,68^. At elevated temperatures, thermal energy overcomes potential energy barriers in the disordered network structure, allowing ions or electrons acting as charge carriers to move more freely and frequently between localized sites. Such thermally driven enhancement mirrors well-established patterns reported in the literature for phosphate-based glasses doped with rare-earth elements, where the incorporation of dopants plays a pivotal role in modulating electrical properties^64,68,69^. Specifically, dopants with comparatively smaller ionic radii, such as Ce^3^⁺ (cerium in its trivalent state), exert a subtle yet significant influence on the conduction mechanism of the glass. The reduced size of these ions leads to a more compact local coordination environment within the phosphate polyhedral, which in turn affects the activation energy required for charge carrier hopping or migration^68,70,71^. Lower activation energies, often resulting from such dopant-induced structural tweaks, promote easier thermal excitation and thereby amplify the overall AC conductivity at higher temperatures^63,68^. This interplay between dopant ionic radius, network distortion, and thermal activation not only dictates the temperature-dependent conductivity profiles but also underscores broader trends in rare-earth-doped phosphate glasses, where similar behaviors have been documented in studies involving spectroscopic and impedance analyses.

Among the various dopants investigated, the incorporation of rare-earth oxides such as CeO_2_, Ho_2_O_3_, and Sm_2_O_3_ leads to a noticeable enhancement in the AC conductivity when compared with the undoped base glass, underscoring the significant role of these additives in modifying the electrical properties of the glass network. This improvement in conductivity is primarily associated with structural changes induced by the dopant ions, particularly the formation of non-bridging oxygen atoms and the presence of modifier ions, which together contribute to an increase in the number of available charge carriers and provide favorable pathways for ion hopping conduction^68,73^. The introduction of rare-earth ions disrupts the continuous glass network, lowers the energy barriers for charge transport, and enhances the mobility of ions under an alternating electric field. Furthermore, the observed differences in AC conductivity among the CeO_2_-, Ho_2_O_3_ -, and Sm_2_O_3_-doped glasses can be explained by variations in the ionic radii of the dopant ions and their distinct influence on the rigidity and compactness of the glass structure. Smaller ionic radii tend to produce a less rigid network with greater structural flexibility, which in turn facilitates easier ion movement and results in higher conductivity values^68,72,73^.

On the other hand side, The AC conductivity of the prepared glasses exhibit a clear increase as the applied frequency rises, which is a hallmark of semiconducting behavior typically observed in disordered glass matrices^19,74,75^. This trend arises primarily from the progressive release of space charges and the dominance of charge carrier hopping mechanisms, which become increasingly prominent at elevated frequencies^19,74–76^. In such amorphous structures, low-frequency conduction is often limited by trapped charges and localized states, but as frequency escalates, these carriers gain sufficient energy to overcome potential barriers, facilitating short-range hops between neighboring sites^19,74–76^. This frequency-dependent response underscores the role of structural disorder in governing electrical transport, aligning with established models for non-crystalline semiconductors where polaronic hopping and interfacial polarization contribute significantly to the observed conductivity enhancement.

Following exposure to gamma irradiation (Fig. 14), the AC conductivity of the undoped and rare-earth doped glasses exhibits a modest decline relative to its pre-irradiation levels. This subtle decrease can be attributed to radiation-induced structural changes, including the compaction of the glass network and the generation of defects, both of which impede the mobility of charge carriers and thus restrict overall charge transport processes^64,76^. Despite this reduction, the fundamental patterns and behaviors in AC conductivity that were evident prior to irradiation remain largely intact, aligning well with documented observations in borate-based glasses and similar irradiated glass systems where such radiation effects lead to comparable minor alterations without disrupting core electrical response trends.

**Fig. 15:**
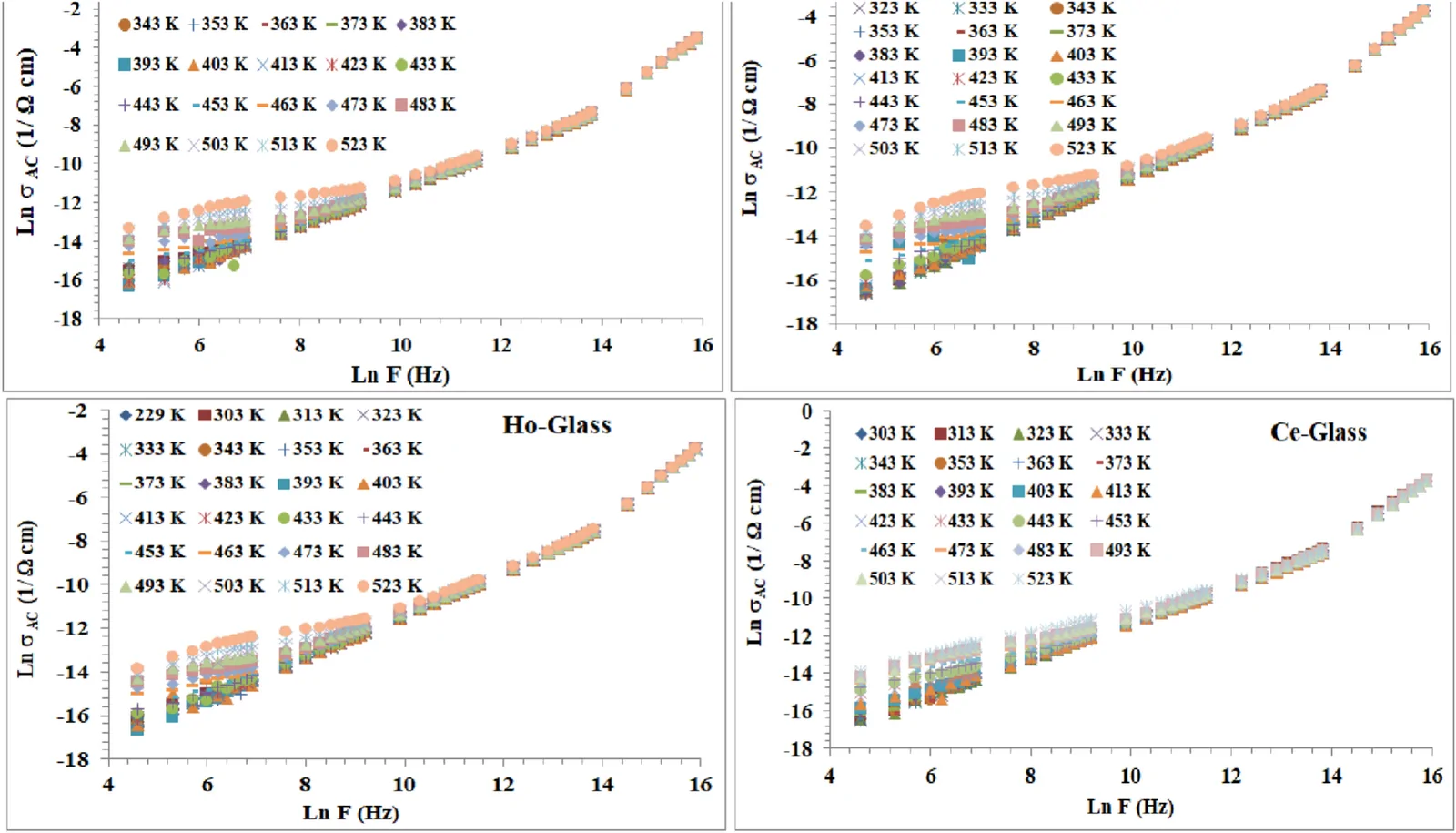
*Ac* conductivity as Ln(σ_*Ac*_) with 1000/T for blank and rare – earths doped glass before gamma irradiation.

**Fig. 16:**
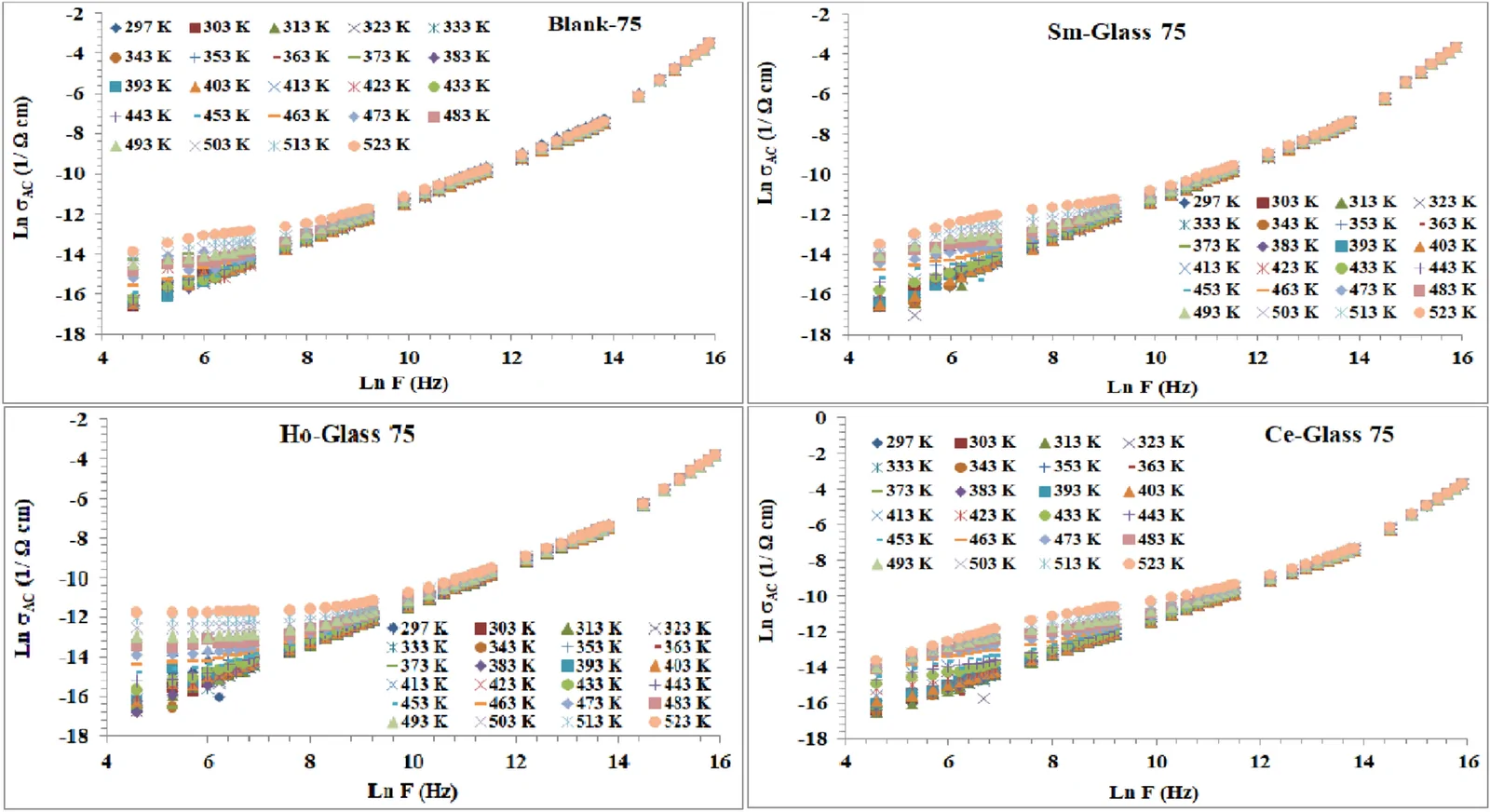
*Ac* conductivity as Ln(σ_*Ac*_) with Frequency at different temperature for blank and rare – earths doped glass after gamma irradiation.

The frequency dependence of the AC conductivity at different temperatures for all glasses studied is shown in Figs. 15 and 16. Note that the AC conductivity changes with low frequencies (100–100,000 Hz), while at high frequencies (up to 100,000 Hz) it has almost the same values. The values of σ_ac_ (ω) for all glass samples were found to obey the general relationship^20,21^

15 $$\upsigma \mathrm{a}\mathrm{c} (\upomega ) = A \upomega \mathrm{s}$$

where ω is the angular frequency, A is the temperature dependence constant and s is the frequency exponent. The frequency exponent (s) values were determined to be between 1.48 and 0.01 in the studied range of temperature. This kind of variation of (s) with temperature has been observed in different TMI-doped phosphate^77^ and borophosphate [80] glasses, where the value of s is greater than 1. The observed variation of (s) with temperature may be due to different contributions from conducting and dielectric losses at different temperatures^77,78^. Three theoretical models exist for explaining the behavior of electrical conductivity.

1. Simple quantum mechanical tunneling (QMT) model
2. Non-overlapping small polaron tunneling (NSPT) model
3. Overlapping large polaron tunneling (OLPT) model.

In the QMT model, the frequency exponent (s) is independent of temperature but varies as a function of frequency. Conversely, within the NSPT model, (s) demonstrates a temperature-dependent behavior, increasing as the temperature rises. In the case of the OLPT model, another polaron tunneling mechanism, the exponent s is dependent on both frequency and temperature, decreasing from unity as the temperature rises. In the CBH model, a temperature-dependent exponent (s) is predicted, wherein (s) decreases with increasing temperature. For large polaron, the exponent (s) continues to decrease with increasing temperature, approaching the value predicted by the QMT model for non-overlapping carriers. For small polaron (SP), (s) exhibits a minimum at a specific temperature and subsequently increases as the temperature rises.

**Fig. 17:**
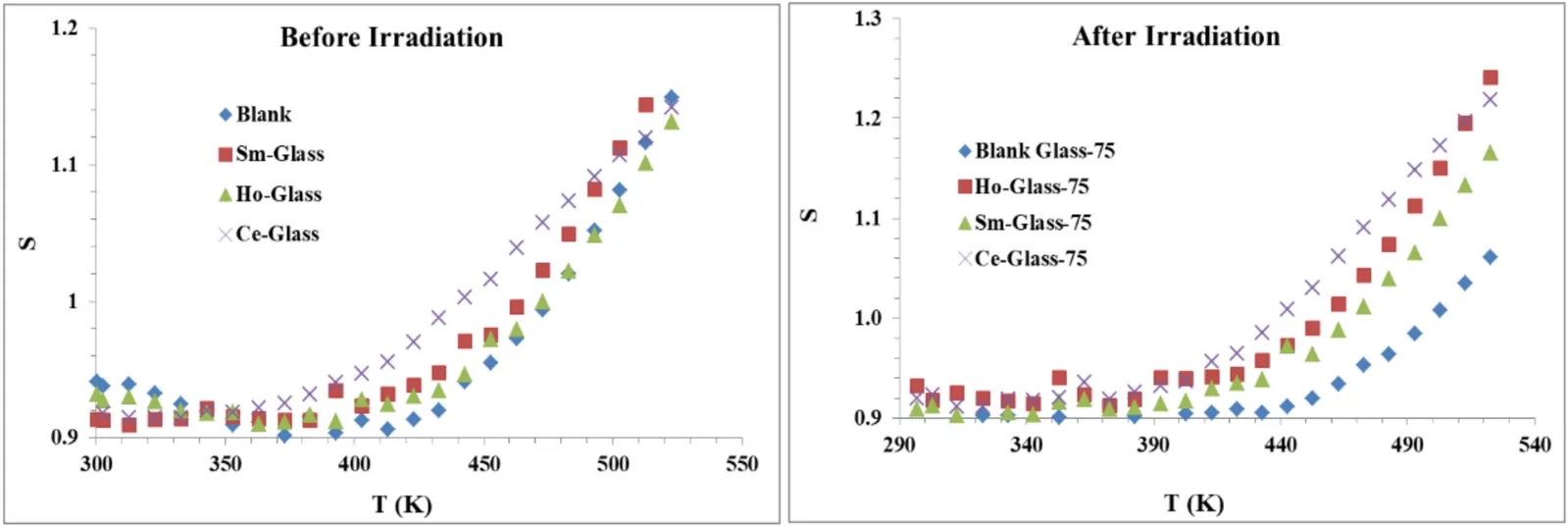
The variation of s values for all glass samples as a function of temperature.

Figure 17 displays the temperature dependence of the frequency exponent (s); obtained from nonlinear fits using Eq. (15) for the present glasses. At low temperatures, s exhibits a minimum at a certain temperature and then increases with temperature. This behavior reflects the presence of a small polaron. In other words, for all the glass of the investigated samples, the value of s increases with increasing temperature, which agrees with the NSPT model.

#### The variation of dielectric properties

**Fig. 18:**
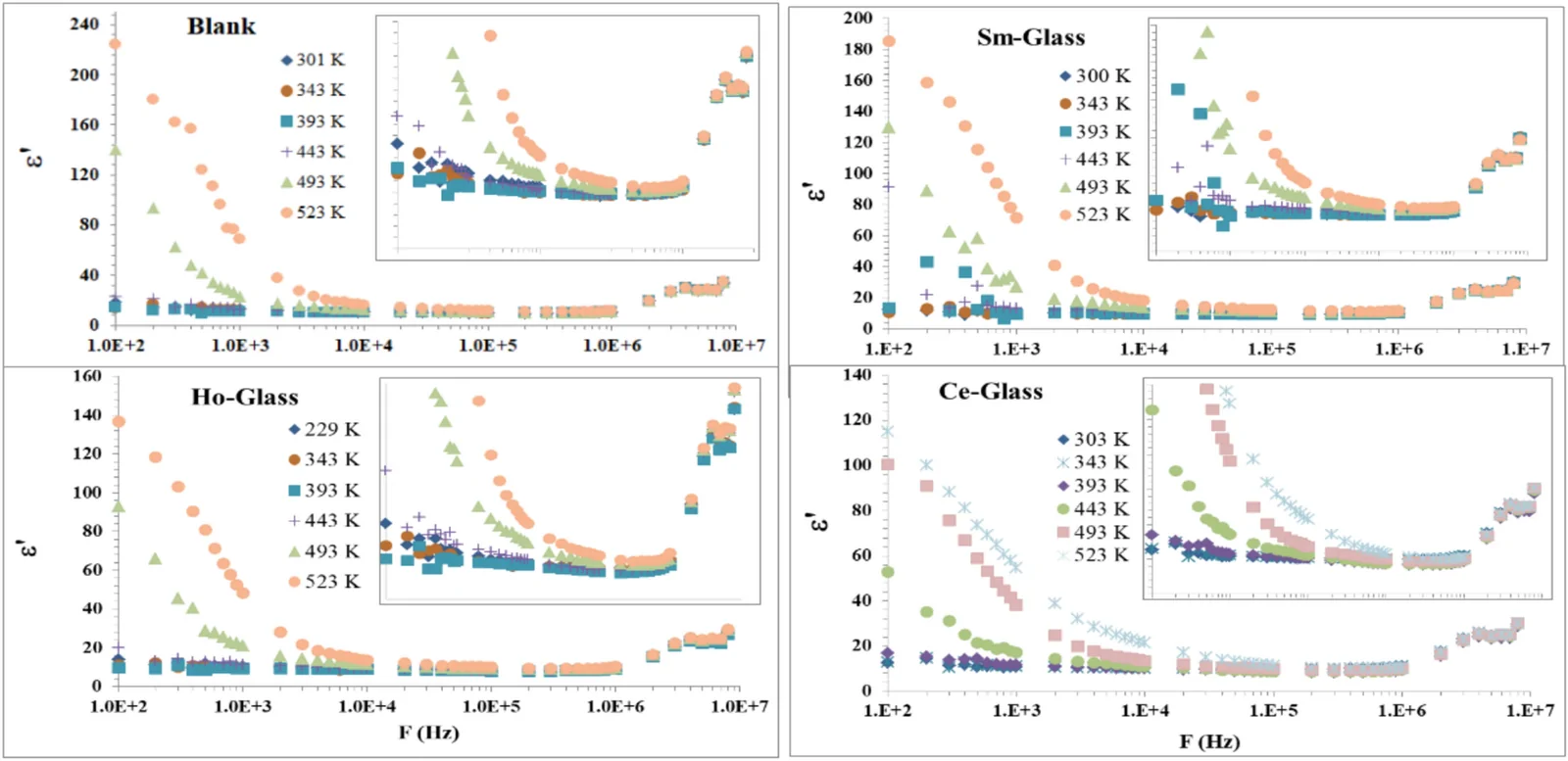
The variation of dielectric constant (ε′) with frequencies at different temperatures for blank and rare -earth doped glass before gamma irradiation.

Figure 18 illustrates the temperature-dependent behavior of the real component of the dielectric permittivity (ε′) across a range of frequencies for glass compositions intentionally doped with varying concentrations of rare-earth. In these synthesized glass samples, the values of ε′ are notably elevated at lower frequencies, a phenomenon primarily ascribed to the buildup of space charge polarization occurring at the interface between the electrodes and the glass specimen^67,79,80^. As the frequency escalates, the influence of these space charges on the overall dielectric response diminishes progressively, leading to a corresponding reduction in ε′. This trend aligns with established principles in dielectric materials, where interfacial polarization effects dominate under low-frequency conditions but become less significant at higher frequencies due to the limited relaxation times of charge carriers.

**Fig. 19:**
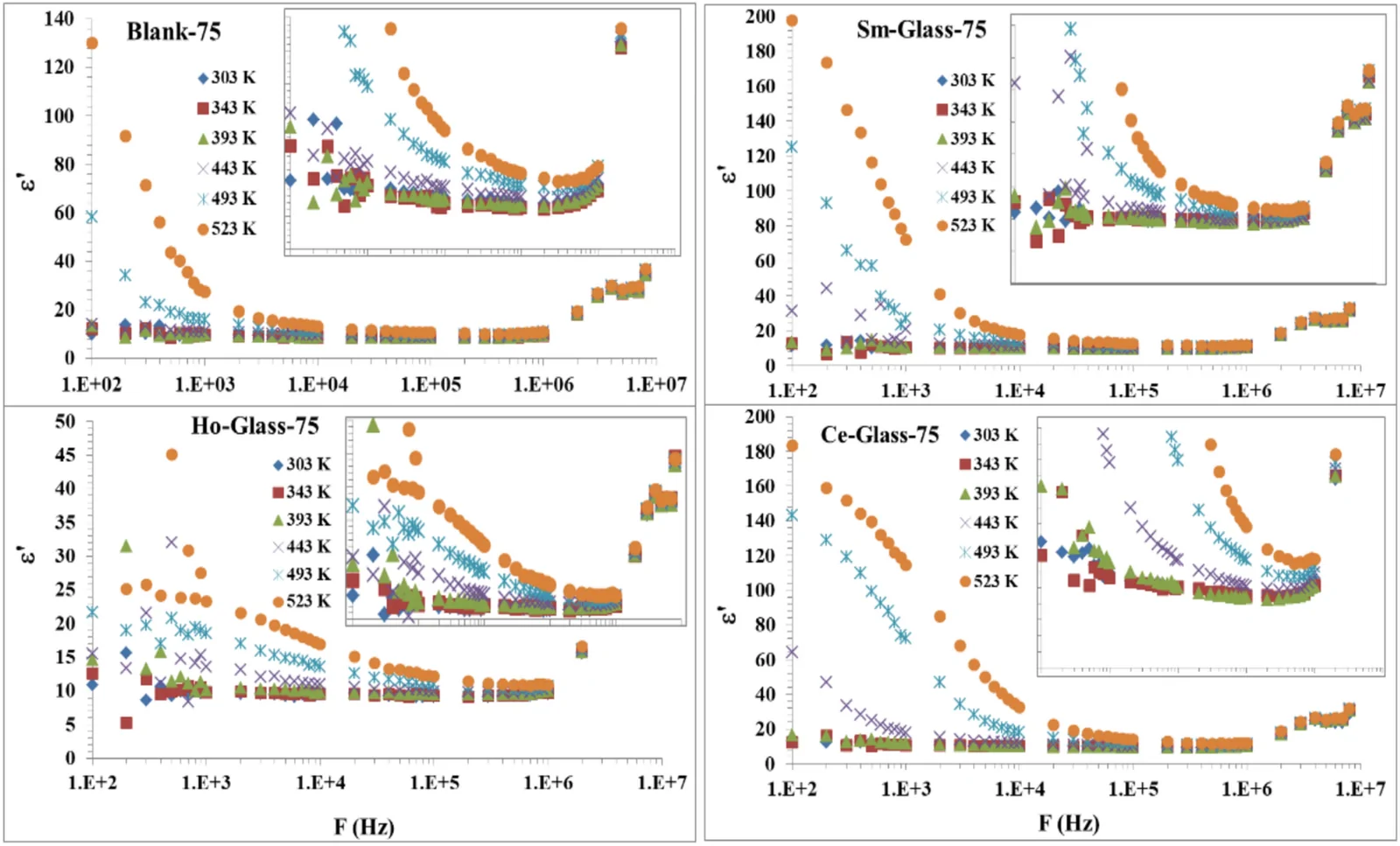
The variation of dielectric constant (ε′) frequencies at different temperatures for blank and rare -earth doped glass after gamma irradiation.

Figure 19 presents the evolution of the dielectric constant in the prepared glass samples following successive exposures to a 75 KGy gamma irradiation dose, revealing only subtle alterations when compared to the pre-irradiation characteristics outlined in the preceding analysis of temperature and frequency dependencies. These minor shifts in the real part of the dielectric permittivity (ε′) confirm the robust structural integrity and inherent shielding efficacy of the rare-earth oxide-doped glasses against high-energy gamma radiation, as the materials demonstrate limited degradation in dielectric performance despite the ionizing effects that could otherwise induce defect formation or charge trapping. Such resilience aligns with observations in radiation-resistant glass systems, where controlled doping enhances cross-linking within the amorphous network, thereby mitigating radiation-induced compaction or bond breakage that might otherwise amplify dielectric dispersion. The negligible changes further corroborate the role of rare-earth ions in stabilizing the glass matrix, preventing significant increases in conductivity or permittivity anomalies typically associated with defect centers post-irradiation.

**Fig. 20:**
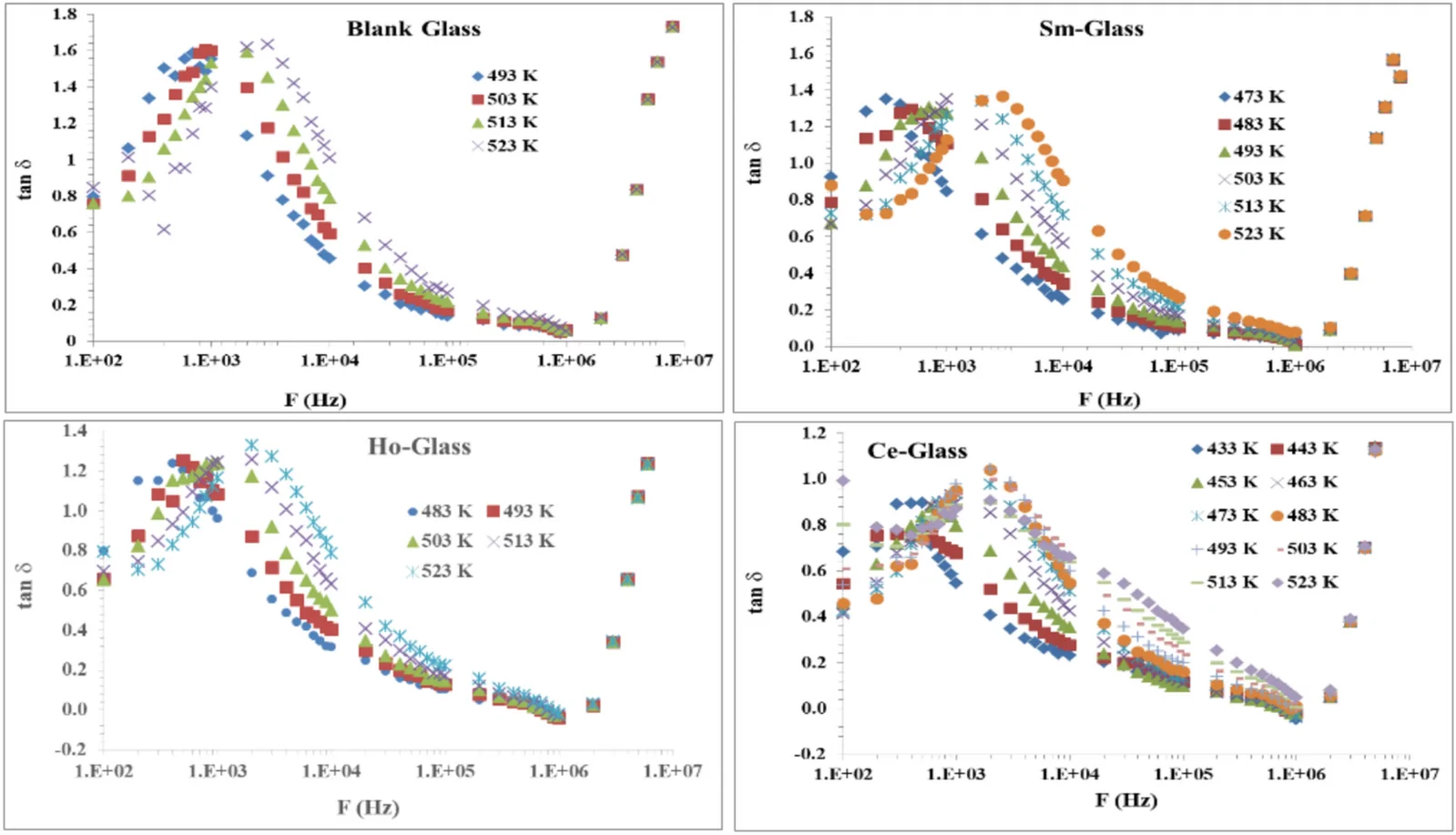
The variation of loss factor (tanδ) with frequencies at different temperature for blank and rare -earth doped glass before gamma irradiation.

**Fig. 21:**
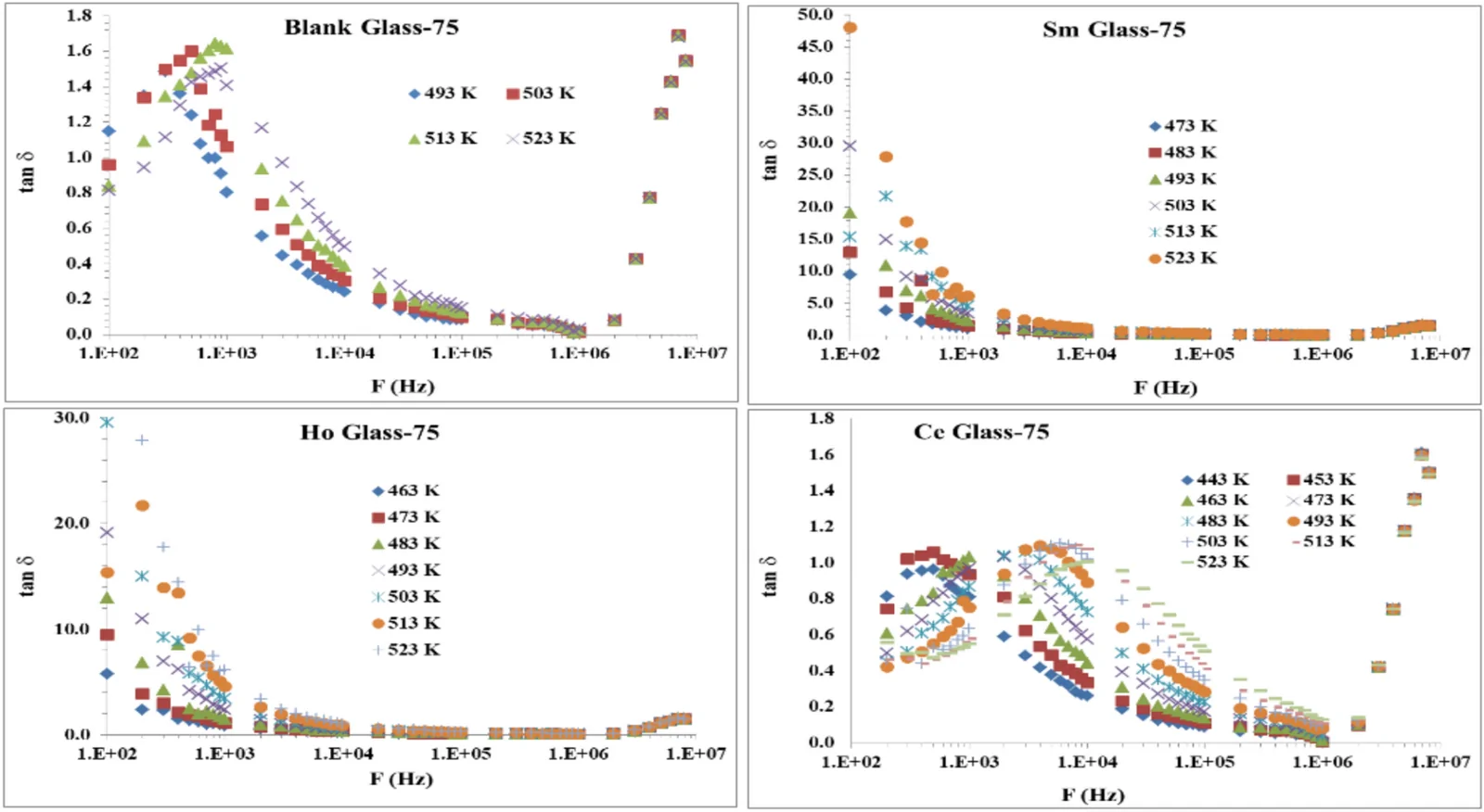
The variation of loss factor (tanδ) with frequencies at different temperature for blank and rare -earth doped glass after gamma irradiation.

The variation of dielectric loss tangent (tanδ) with frequency at different temperatures for all glasses is shown in Figs. 20 and 21. A single relaxation peak was observed for all samples before irradiation, whereas after irradiation, it was present only in the blank sample and those doped with Ce^3^⁺.

**Fig. 22:**
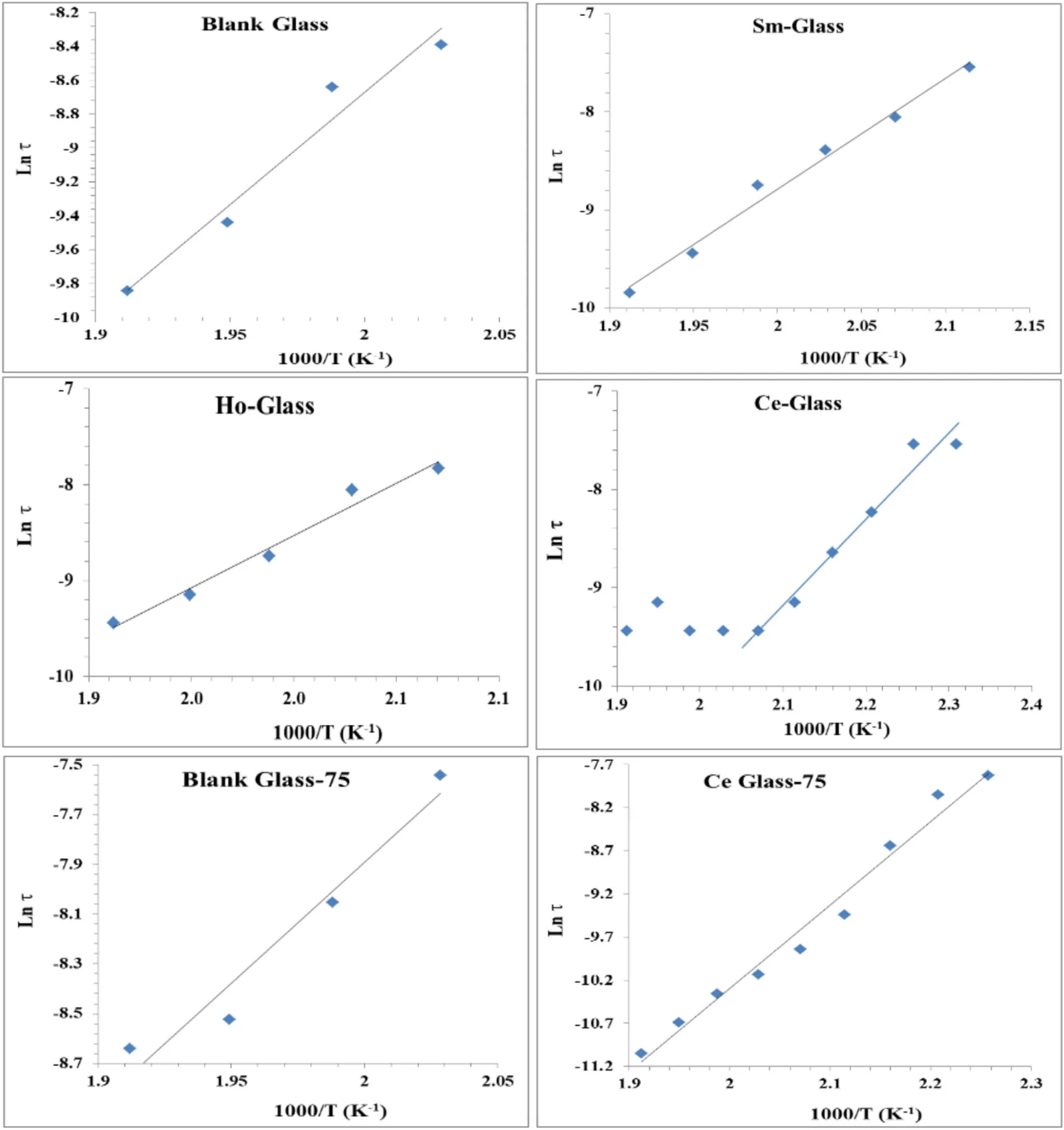
Variation of lnτ with 1000/T for the blank and rare – earths doped glass before and after gamma irradiation.

The maximum shifts to higher frequencies as the temperature increases, indicating the relaxation nature of the dielectric loss in these glasses. The value of tan δ is inversely proportional to the frequency, as described by the Deb equation. The Debye relaxation equation states that the value of ε’ decreases as the frequency a ω increase, which is given simply by:

16 $${\varepsilon }^{\prime}={\varepsilon}_{\infty }+\frac{{\varepsilon}_{0}-{\varepsilon}_{\infty }}{1+{\omega }^{2}{\tau}_{1}^{2}}$$

  where τ is the relaxation time, ε_0_ is the permittivity of free space, and ω is the frequency of the input signal. The decrease in ε′ with increasing frequency indicates the anomalous dispersion of the relative permittivity at low and intermediate frequencies, while the variation of ε″ with frequency reflects the relaxation absorption of the dielectric. The relationship between ε ‘ and ε ‘‘ is defined as a loss tangent, tan δ = ε ‘‘/ε‘. The maximum value of tan δ is attained when ωτ₁ = 1; the corresponding frequency f₀ = 1/(2πτ₁) is referred to as the critical frequency, while tan δ reaches its peak at ωτ = 1. The temperature dependence of the relaxation time τ expressed as an Arrhenius formula:

17 $$\tau ={\tau}_{0}{e}^{E/KT}$$

where *E* is the activation energy of the relaxation process, τ_0_ is a constant quantity and *K* is the Boltzmann constant. To obtain the value of E for a relaxation mechanism, the maximum tan δ_m_ value must be defined first. Therefore, Figs. 20 and 21 show the relationship between tanδ and applied frequency (F) at different temperatures. From Figs. 20 and 21, we can determine the frequency for the maximum, tan δm, and calculate the relaxation time τ, where ωτ = 1 and ω = 2πF. The relationship between lnτ and 1000/T is a straight line (as in Fig. 22), to obtain the value of E for the relaxation mechanism. This means that there is only one type of conductivity mechanism, as previously explained. The activation energy values for the relaxation mechanisms calculated from Figs. 20 and 21 are listed in Table 5. In general, the value of the activation energy indicates the degree of freedom of the charge carrier. In other words, a high value of the activation energy corresponds to high conductivity.

**Table 5:** The activation energy E (eV)for the relaxation mechanism obtained from the relationship between lnτ and 1000/T shown in Fig. 19 before and after gamma irradiation.

Sample	Before	After
Blank- glass	1.1457	0.8399
Ho-glass	0.9401	-
Sm-glass	0.9744	-
ce-glass	0.6854	0.8337

### Structural investigation via FTIR absorption spectra

**Fig. 23:**
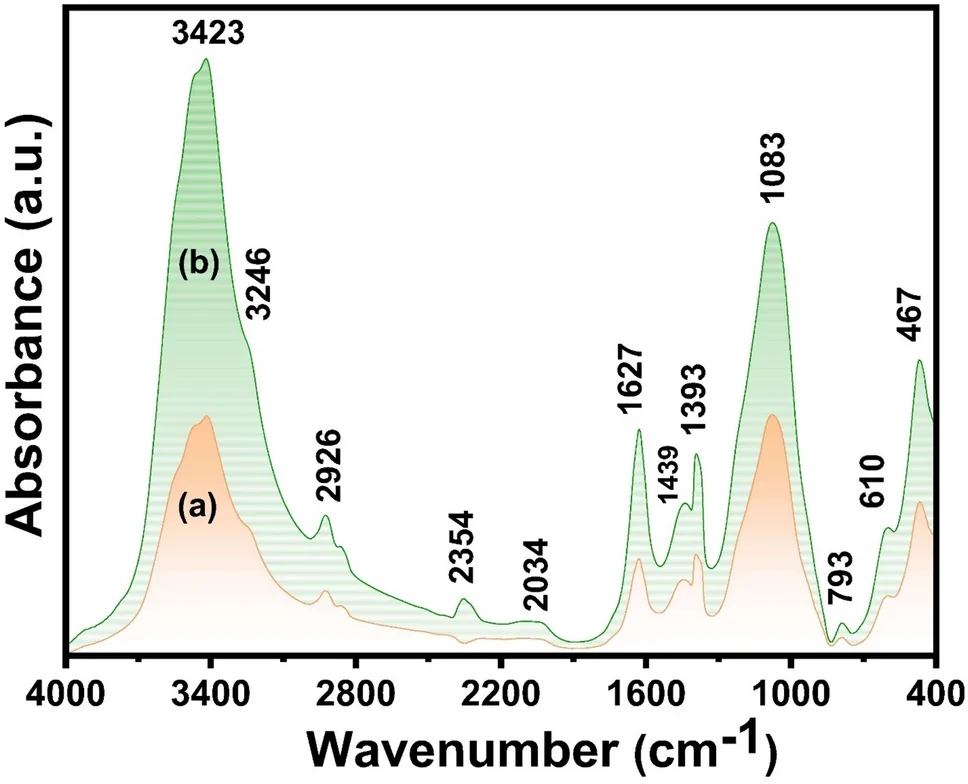
FTIR absorption spectra of the prepared base glass where (**a**) before and (**b**) after 75 KGy dose of gamma irradiation.

Figures 23(a) and (b) present the FTIR spectra of prepared glasses before and after exposure to 75 kGy γ-irradiation, respectively. The spectra exhibit several characteristic absorption bands corresponding to the vibrational modes of borate, phosphate, and alumino-oxygen structural units. Comparison of the spectra before and after irradiation indicates that the glass network remains largely preserved, with only minor structural modifications induced by γ-rays.

Before gamma irradiation, the absorption band observed at 467 cm^−1^ is attributed to the bending vibrations of O–P–O and O-B-O linkages as well as lattice vibrations of the glass network^44^. The band at 610 cm^−1^ is assigned to the bending vibration of phosphate groups and B-O-B bridging oxygen bonds^7,44^. The presence of these low-frequency bands confirms the formation of a connected borophosphate glass network. The band located at 793 cm^−1^ is characteristic of the symmetric stretching vibration of B-O bonds in tetrahedral BO_4_ units and/or P-O-P symmetric stretching vibrations^25,27^. The appearance of this band suggests the coexistence of borate and phosphate structural units within the glass matrix. A strong absorption band at 1083 cm^−1^ corresponds to the asymmetric stretching vibrations of PO_4_ tetrahedra (P-O bonds) and B-O stretching vibrations in BO_4_ units. This band is considered a principal feature of borophosphate glasses and reflects the connectivity of the phosphate network. The bands observed at 1393 cm^−1^ and 1439 cm^−1^ are assigned to the asymmetric stretching vibrations of B-O bonds in trigonal BO_3_ units^27,35^. These bands indicate the presence of borate groups with threefold coordination and suggest the coexistence of both BO_3_ and BO_4_ structural units in the glass network.

The absorption band at 1627 cm^−1^ is attributed to the bending vibration (H–O-H) of molecular water adsorbed on the glass surface. The weak bands appearing at 2034 cm^−1^ and 2354 cm^−1^ are generally associated with atmospheric CO_2_ and combination or overtone vibrations, rather than intrinsic structural units of the glass. Similarly, the weak band at 2926 cm^−1^ may originate from O–H-related combination bands or trace organic residues introduced during sample preparation^27,44^.

The broad absorption bands centered at 3246 cm^−1^ and 3423 cm^−1^ are assigned to the stretching vibrations of hydroxyl (O–H) groups and hydrogen-bonded water molecules. The broad nature of these bands indicates the existence of hydrogen-bonded hydroxyl species within the glass network or adsorbed moisture on the sample surface^25,27^.

After exposure to 75 kGy γ-irradiation (Fig. 23b), the FTIR spectrum retains essentially the same absorption bands as the unirradiated sample, indicating that γ-irradiation does not produce significant changes in the fundamental glass structure. However, slight variations in band intensity and/or minor shifts in peak positions may occur due to radiation-induced rearrangement of the glass network. Gamma irradiation can generate electron–hole pairs and defect centers, leading to localized breaking and reforming of B-O-B, P-O-P, and B-O-P bridges. Such rearrangements may slightly modify the relative populations of BO_3_ and BO_4_ units and alter the distribution of non-bridging oxygen atoms without causing crystallization or major structural degradation. Consequently, the persistence of the characteristic FTIR bands after irradiation demonstrates the high structural stability of the prepared sodium borophosphate glass. The observed spectral changes are therefore attributed to local structural relaxation and defect formation rather than significant alterations in the overall glass network.

## Conclusions

A series of undoped and rare-earth-doped borophosphate glass systems containing CeO_2_, Ho_2_O_3_, and Sm_2_O_3_ was successfully synthesized using the melt quenching technique followed by annealing. Structural, optical, luminescence, and electrical studies were performed to evaluate the influence of rare-earth incorporation and gamma irradiation on the physical properties of these glasses. The results confirm that both doping and irradiation induce only subtle modifications while preserving the overall stability and functional performance of the glass network. X-ray diffraction analysis verified the amorphous nature of all prepared samples, as indicated by the absence of sharp crystalline peaks and the presence of broad halos. This confirms that the addition of rare-earth oxides does not promote crystallization and maintains the disordered glassy structure, which is favorable for optical uniformity and reduced scattering losses. UV–visible absorption measurements revealed strong ultraviolet absorption for the undoped glass, with characteristic bands around 265 and 330 nm. After exposure to a gamma dose of 75 kGy, the absorption profile remained largely unchanged, except for a slight decrease in peak intensity, indicating minor radiation-induced defect formation without significant alteration of the electronic structure. Similar behavior was observed in CeO_2_-doped glasses, which exhibited stable UV absorption peaks at approximately 220, 260, and 327 nm before and after irradiation. This stability demonstrates the radiation-resistant nature of the glass matrix. Ho^3+^- and Sm^3+^-doped glasses displayed multiple well-defined absorption bands across the UV–visible–NIR regions, corresponding to intra-4f. electronic transitions of the rare-earth ions. The persistence of these transitions after irradiation highlights the effective shielding provided by the amorphous host. In particular, Sm_2_O_3_-doped glasses exhibited a broad spectral response, underscoring their suitability for photonic and optoelectronic applications. Photoluminescence studies showed characteristic excitation and emission features for all doped samples. CeO_2_-doped glass exhibited strong near-UV excitation and visible emission, while Ho_2_O_3_- and Sm_2_O_3_-doped glasses displayed sharp emission peaks corresponding to green and reddish-orange emissions, respectively. Although gamma irradiation caused a reduction in emission intensity, the emission wavelengths remained unchanged, suggesting that non-radiative defect centers increased without disrupting the intrinsic luminescent mechanisms. CIE 1931 chromaticity analysis confirmed distinct and tunable color emissions for each dopant. Electrical investigations revealed temperature-dependent AC and DC conductivity behavior typical of ionic conduction in glassy systems. Conductivity initially decreased with temperature, reaching a minimum near 384 K, followed by a slight increase at higher temperatures. Dielectric permittivity showed high values at low frequencies due to space charge polarization. Only minor changes in electrical properties were observed after irradiation. Overall, the slight variations in optical and electrical properties are attributed to structural modifications such as the formation of non-bridging oxygen atoms and the presence of modifier ions, which enhance charge carrier mobility. The activation energy reflects the mobility of charge carriers in the material, where lower activation energy corresponds to higher electrical conductivity and higher activation energy indicates reduced conductivity. These findings demonstrate that rare-earth-doped glasses exhibit excellent structural, optical, and electrical stability under gamma irradiation, making them promising candidates for radiation-resistant optical and electronic applications.

## Data Availability

The datasets used and/or analysed during the current study available from the corresponding authors on reasonable request.

## References

[1] Matzkeit, Y. H. et al. Borophosphate glasses: Synthesis, characterization and application as catalyst for bis(indolyl) methanes synthesis under greener conditions. *J. Non-Cryst. Solids* **498**, 153–159 (2018).

[2] Massera, J. et al. Processing and characterization of novel borophosphate glasses and fibers for medical applications. *J. Non-Cryst. Solids* **425**, 52–60 (2015).

[3] Ramakrishna, P. et al. Effect of U on the photoluminescence of Pr and structural properties of U/Pr doped and co-doped Li_2_O-ZnO-SrO borophosphate glass. *Opt. Mater.* **134**, 113121 (2022).

[4] Karki, S. et al. Physical, optical and luminescence properties of the Dy^3+^-doped barium borophosphate glasses. *J. Non-Cryst. Solid.* **521**, 119483 (2019).

[5] Griebenow, K. et al. Structure and fluorescence properties of Dy-doped alkaline-earth borophosphate glasses. *Int. J. Appl. Glass Sci.* **12**, 472–484 (2021).

[6] Dinca, M. C. et al. Magneto-optical properties of borophosphate glasses co-doped with Tb^3+^ and Dy^3+^ ions. *J. Non-Cryst. Solid.* **568**, 120967 (2021).

[7] Koudelka, L. & Mošner, P. Study of the structure and properties of Pb–Zn borophosphate glasses. *J. Non-Cryst. Solids* **293–295**, 635–641 (2001).

[8] Anantha, P. S. & Hariharan, K. Structure and ionic transport studies of sodium borophosphate glassy system. *Mater. Chem. Phys.* **89**, 428–437 (2005).

[9] Nakayama, S. et al. Influence of rare earth additives and boron component on electrical conductivity of sodium rare earth borate glasses. *Ceram. Int.* **36**, 2323–2327 (2010).

[10] Ravi Kumar, A. V., Apparao, B. & Veeraiah, N. Dielectric properties of LiF–B_2_O_3_ glasses doped with certain rare earth ions. *Bull. Mater. Sci.* **21**, 341–347 (1998).

[11] Sołtys, M., Górny, A., Pisarska, J. & Pisarski, W. A. Electrical and optical properties of glasses and glass-ceramics. *J. Non-Cryst. Solid.* **498**, 352–363 (2018).

[12] Yongsiri, P., Sirisoonthorn, S. & Pengpat, K. Effect of Er_2_O_3_ dopant on electrical and optical properties of potassium sodium niobate silicate glass-ceramics. *Mater. Res. Bull.* **69**, 84–91 (2015).

[13] Zavadil, J. et al. Investigation of electrical and optical properties of Ge–Ga–As–S glasses doped with rare-earth ions. *J. Non-Cryst. Solid.* **377**, 85–89 (2013).

[14] Eftimie, M., Filip, A. V., Danescu, C. B., Nitescu, A. & Sava, B. A. Obtaining and conductive properties of a vanadate-borate-phosphate glass. *Sci. Rep.* **13**, 16054 (2023). 37749211 10.1038/s41598-023-43302-8 PMC10519944

[15] Biswas, D. et al. Effect of AgI doping on electrical conductivity and dielectric relaxation in silver phosphate glass nanocomposite systems. *Physica B* **602**, 412486 (2021).

[16] Schubert, E. F. *Light Emitting Diodes* 2nd edn. (Cambridge University Press, 2006).

[17] Malacara, D. *Color Vision and Colorimetry: Theory and Applications* 2nd edn. (SPIE Press, 2011).

[18] Mortimer, R. J. & Varley, T. S. Displays. *Displays* **35**, 32 (2011).

[19] Fayek, M. K., Ata-Allah, S. S., Roumaih, K. & Ismail, S. Thermoelectric power properties of Zn-substituted Cu–Ga spinel ferrites. *Mater. Lett.* **63**, 1010–1012 (2009).

[20] Roumaih, K. Electrical and magnetic properties of MF/CuAl nanocomposites. *Z. Naturforsch. A* **79**, 517–541 (2024).

[21] Roumaih, K. Effect of temperature on the dielectric and magnetic properties of NiFe_2_O_4_@MgFe_2_O_4_ and ZnFe_2_O_4_@MgFe_2_O_4_ core-shell. *Phys. Scr.* **96**, 125809 (2021).

[22] Duffy, J. A. Charge transfer spectra of metal ions in glass. *Phys. Chem. Glasses* **38**, 289–294 (1997).

[23] Ehrt, D., Ebeling, P. & Natura, U. UV transmission and radiation-induced defects in phosphate and fluoride-phosphate glasses. *J. Non-Cryst. Solid.* **263–264**, 240–250 (2000).

[24] Möncke, D. & Ehrt, D. Irradiation induced defects in glasses resulting in the photoionization of polyvalent dopants. *Opt. Mater.* **25**, 425–437 (2004).

[25] Marzouk, M. A., ElBatal, F. H., Eisa, W. H. & Ghoneim, N. A. Comparative spectral and shielding studies of binary borate glasses with the heavy metal oxides SrO, CdO, BaO, PbO or Bi_2_O_3_ before and after gamma irradiation. *J. Non-Cryst. Solid.* **387**, 155–160 (2014).

[26] Leonelli, C., Lusvardi, G., Malavasi, G., Menabue, L. & Tonelli, M. Synthesis and characterization of cerium-doped glasses and *in vitro* evaluation of bioactivity. *J. Non-Cryst. Solid.* **316**, 198–216 (2003).

[27] Marzouk, M. A., Ali, I. S. & ElBatal, H. A. Optical, FT infrared and photoluminescence spectra of CeO₂-doped Na₂O–ZnO–B₂O₃ host glass and effects of gamma irradiation. *J. Non-Cryst. Solid.* **485**, 14–23 (2018).

[28] Cotton, F. A., Wilkinson, G., Murillo, C. A. & Bochmann, M. *Advanced Inorganic Chemistry* 6th edn. (John Wiley-Interscience, 1999).

[29] Das Mohapatra, G. K. A spectroscopic study of Ce^3^⁺ ion in calcium metaphosphate glass. *Phys. Chem. Glass.* **39**, 50–55 (1998).

[30] Mohapatra, S. K., Khan, S., Chakraborty, S., Maharana, H. S. & Annapurna, K. Ho^3+^-activated calcium zinc silico-aluminate glass for 2 μm and green light emission. *Mater. Today*10.2139/ssrn.4483780 (2023).

[31] Karmakar, B. & Annapurna, K. Blue, green and red upconversions in Ho₂O₃-doped fluorophosphate glasses. *J. Non-Cryst. Solid.* **353**, 1377–1382 (2007).

[32] Carnall, W. T., Fields, P. R. & Rajnak, K. Electronic energy levels in the trivalent lanthanide aquo ions. I. Pr^3+^, Nd^3+^, Pm^3+^, Sm^3+^, Dy^3+^, Ho^3+^, Er^3+^ and Tm^3+^. *J. Chem. Phys.* **49**, 4424–4442 (1968).

[33] Maheswari, T., Naresh, V., Ramaraghavulu, R., Rudramadevi, B. H. & Buddhudu, S. Studies on thermal and spectral properties of Ho^3^⁺: Lithium borate glasses. *Int. J. Eng. Res. Technol.* **3**(7), 574–580 (2014).

[34] Babu, K. V., Kumar, K. N. & Rao, A. S. Studies on spectral and photoluminescence properties of Sm^3+^ ions in sodium lead alumino borosilicate glasses. *J. Emerg. Technol. Innov. Res.* **5**(11), 36–48 (2018).

[35] Sundari, S. S., Marimuthu, K., Sivraman, M. & Babu, S. S. Composition dependent structural and optical properties of Sm^3+^-doped sodium borate and sodium fluoroborate glasses. *J. Lumin.* **130**, 1313–1319 (2010).

[36] Sudhakar, K. S. V., Srinivasa Reddy, M., Srinivasa Rao, L. & Veeraiah, N. Influence of modifier oxide on spectroscopic and thermoluminescence characteristics of Sm^3+^ ion in antimony borate glass system. *J. Lumin.* **128**, 1791–1798 (2008).

[37] Mott, N. F. & Davis, E. A. *Electronic Processes in Non-Crystalline Materials* 2nd edn. (Clarendon Press, 1979).

[38] Urbach, F. The long-wavelength edge of photographic sensitivity and of the electronic absorption of solids. *Phys. Rev.* **92**, 1324–1324 (1953).

[39] Marzouk, M. A., Ali, I. S., Ali, M. M. & Hamdy, Y. M. Laser compatible emission and enhanced microhardness in Y₂O₃- and Nd₂O₃-doped borosilicate glasses incorporating construction demolition waste. *Opt. Quantum Electron.* **58**, 314 (2026).

[40] Was, G. S. *Fundamentals of Radiation Materials Science: Metals and Alloys* 2nd edn. (Springer, 2017).

[41] Pelant, I. & Valenta, J. *Luminescence Spectroscopy of Semiconductors* (Oxford University Press, 2012).

[42] Lai, Y. et al. Investigation of gamma-ray induced optical property changes in non-doped and Ce-doped lithium-rich oxide glass. *Radiat. Phys. Chem.* **179**, 109272 (2021).

[43] Ali, I. S. Enhancing the luminescent properties of strontium phosphate glass via controlled crystallization and rare earth dopants. *J. Mater. Sci.: Mater. Electron.* **35**, 1767 (2024).

[44] ElBatal, F. H., Marzouk, M. A., ElBatal, H. A. & Hamdy, Y. M. Behavior of bismuth ions in the two glassy systems of 50LiF–50P₂O₅ and 50LiF–50B₂O₃ (mol%) assessed by optical, FTIR and photoluminescence spectra in addition to thermal expansion properties. *Opt. Quantum Electron.* **55**, 217 (2023).

[45] Zheng, L. H. et al. Luminescence properties of Ce^3+^-doped lithium borophosphate glasses and their correlations with the optical basicity. *J. Non-Cryst. Solid.* **403**, 1–4 (2014).

[46] Marzouk, M. A. & Ghoneim, N. A. Gamma irradiation and crystallization effects on the photoluminescence properties of soda lime fluorophosphate host glass activated with Ce^4+^, Dy^3+^ or Pr^3+^ ions. *Radiat. Phys. Chem.* **174**, 108893 (2020).

[47] Hobbs, L. W., Sreeram, A. N., Jesurum, C. E. & Berger, B. A. Structural freedom, topological disorder, and the irradiation-induced amorphization of ceramic structures. *Nucl. Instrum. Method. Phys. Res. B* **116**, 18–25 (1996).

[48] Mandal, S., Manna, S., Biswas, K., Nag, S. & Ambade, B. Effect of gamma ray irradiation on optical and luminescence properties of CeO_2_-doped bismuth glass. *Ceram. Int.* **49**, 23878–23886 (2023).

[49] Mahamuda, S., Swapna, K., Packiyaraj, P., Rao, A. S. & Prakash, G. V. Visible red, NIR and mid-IR emission studies of Ho^3^⁺-doped zinc alumino bismuth borate glasses. *Opt. Mater.* **36**, 362–371 (2013).

[50] Linganna, K., Rathaiah, M., Venkatramu, V. & Jayasankar, C. K. Spectroscopic properties of Ho^3+^-doped K-Sr–Al phosphate glasses. *Appl. Phys. A* **115**, 689–696 (2014).

[51] Reddy Prasad, V., Damodaraiah, S. & Ratnakaram, Y. C. Optical spectroscopy and luminescence properties of Ho^3+^-doped zinc fluorophosphate (ZFP) glasses for green luminescent device applications. *Opt. Mater.* **78**, 63–71 (2018).

[52] Hussain, N. S. et al. Absorption and emission properties of Ho^3+^-doped lead–zinc–borate glasses. *Thin Solid Film.* **515**, 318–325 (2006).

[53] Suhasini, T., Jamalaiah, B. C., Chengaiah, T., Kumar, J. S. & Moorthy, L. R. An investigation on visible luminescence of Ho^3^⁺-activated LBTAF glasses. *Phys. B* **407**, 523–527 (2012).

[54] Marzouk, M. A., Hamdy, Y. M., ElBatal, H. A. & Ezz-ElDin, F. M. Study on the reducing effect of γ-irradiation on Sm^3+^-doped LiAl fluorophosphate glasses through optical, structural and luminescence analysis. *J. Mater. Sci. Mater. Electron.* **29**, 1399–1411 (2018).

[55] Marzouk, M. A., Hamdy, Y. M. & ElBatal, H. A. Photoluminescence and spectral performance of manganese ions in zinc phosphate and barium phosphate host glasses. *J. Non-Cryst. Solid.* **458**, 1–14 (2017).

[56] Seshadri, M., Rao, K. V., Rao, J. L. & Ratnakaram, Y. C. Spectroscopic and laser properties of Sm^3+^-doped different phosphate glasses. *J. Alloys Compd.* **476**, 263–270 (2009).

[57] Reza Dousti, M. et al. Structural and optical study of samarium-doped lead zinc phosphate glasses. *Opt. Commun.* **300**, 204–209 (2013).

[58] Dieke, G. H. *Spectra and Energy Levels of Rare Earth Ions in Crystals* (Wiley, 1968).

[59] Marzouk, M. A., Hamdy, Y. M., ElBatal, H. A. & EzzElDin, F. M. Photoluminescence and spectroscopic dependence of fluorophosphate glasses on samarium ions concentration and the induced defects by gamma irradiation. *J. Lumin.* **166**, 295–303 (2015).

[60] Fayad, A. M., Ouis, M. A., Morsi, R. M. M. & Elwan, R. L. Enhancement of electrical conductivity associated with non-bridged oxygen defects in molybdenum phosphate oxide glass via doping of SrO. *Sci. Rep.* **13**, 18321 (2023). 37884640 10.1038/s41598-023-45333-7 PMC10603061

[61] Barczyński, R. J., Król, P. & Murawski, L. AC and DC conductivities in V_2_O_5_–P_2_O_5_ glasses containing alkaline ions. *J. Non-Cryst. Solid.* **356**, 1965–1967 (2010).

[62] Osman, N. F., Elokr, M. M., Soliman, L. I. & Zayed, H. A. Synthesis and study of electrical properties of Li₂O-modified P₂O₅–ZnO–Na₂O glasses. *J. Sci. Res. Sci.* **36**, 163–183 (2019).

[63] Marzouk, M. A. & ElBatal, H. A. Investigation of photoluminescence and spectroscopic properties of Sm^3+^-doped heavy phosphate glasses before and after gamma irradiation. *Appl. Phys. A* **127**, 70 (2021).

[64] El Batal, F. H. & Ashour, A. H. Effect of gamma irradiation on the electrical conductivity of ternary borate glasses. *Mater. Chem. Phys.* **77**, 677–686 (2003).

[65] Roumaiha, K., Kaiser, M., ElBatal, F. H. & Ali, I. S. Transport properties of lead phosphate glass doped by cobalt, vanadium and chromium oxides. *Philos. Mag.* **91**, 3830–3843 (2011).

[66] Mokhtar, K., Mohamed, K., Lakhdar, G., Sébastien, B. & Hama, A. Electrical conductivity and dielectric properties of rare earth ions (Ce^3+^, Pr^3+^ and Eu^3+^) doped in zinc sodium phosphate glass. *J. Non-Cryst. Solid.* **567**, 120933 (2021).

[67] Anjaiah, J. et al. Influence of rare-earth ion doping on dielectric properties of lithium zinc borate glasses. *Opt. Mater.* **131**, 112718 (2022).

[68] Paul Dhinakaran, A., Vinothkumar, P., Praveenkumar, S. & Mohapatra, M. The effect of Ce^3^⁺ ions on the optical and radiation shielding properties in Ba–Sn borophosphate glass. *Radiat. Phys. Chem.* **226**, 112357 (2025).

[69] Zhou, R., Calahoo, C., Ding, Y. & Wondraczek, L. Role of Ag^+^ ions in determining Ce^3+^ optical properties in fluorophosphate and sulfophosphate glasses. *ACS Omega* **6**, 30093–30107 (2021). 34778681 10.1021/acsomega.1c04933 PMC8582266

[70] Sendova, M., Jiménez, J. A. & Honaman, C. Rare earth-dependent trend of the glass transition activation energy of doped phosphate glasses: Calorimetric analysis. *J. Non-Cryst. Solid.* **450**, 18–22 (2016).

[71] Kumari, C., Chhoker, S. & Sharma, P. Effect of rare earth dopant on the AC conductivity and dielectric study of GeSbSe chalcogenide glasses. *J. Non-Cryst. Solid.* **616**, 122439 (2023).

[72] Poria, K., Parmar, R., Dhankhar, S. & Kundu, R. S. Electrical conductivity and hopping conduction mechanism by VRH model in halide-modified tellurite glasses. *Solid State Sci.* **148**, 107442 (2024).

[73] Malge, A., Sankarappa, T., Sujatha, T., Ashwajeet, J. S. & Devidas, G. B. Thermoelectric power properties of Zn-substituted Cu-Ga spinel ferrites. *AIP Conf. Proc.* **2161**, 020026 (2019).

[74] Jonscher, A. K. *Dielectric Relaxation in Solids* (Chelsea Dielectrics Press, 1983).

[75] Elliott, S. R. *Physics of Amorphous Materials* 2nd edn. (Longman Scientific & Technical, 1983).

[76] Luo, Y. & Flewitt, A. J. Revisiting multiple trapping and release charge transport in amorphous semiconductors exemplified by hydrogenated amorphous silicon. *J. Non-Cryst. Solid.* **654**, 123436 (2025).

[77] Cutroni, M. et al. Frequency and temperature dependence of AC conductivity of vitreous silver phosphate electrolytes. *Solid State Ionics* **90**, 167–174 (1996).

[78] Kumar, B. V., Sankarappa, T., Kumar, S., Kumar, M. P., Sadashivaiah, P. J. & Reddy, R. R. *Physica B: Condens. Matter* **404**, 3487-3491 (2009).

[79] Kingery, W. D., Bowen, H. K. & Uhlmann, D. R. *Introduction to Ceramics* 2nd edn. (John Wiley & Sons, 1975).

[80] Al Kiey, S. A., Abdel-Hameed, S. A. M. & Marzouk, M. A., Influence of transition metals on the development of semiconducting and low thermal expansion TiO_2_-borosilicate glasses and glass ceramics. *Silicon* **16**, 2945–2953 (2024).

